# Supplementary material for: Dietary Habits, Residential Air Pollution, and Chronic Obstructive Pulmonary Disease
Source: Nutrients. 2025 Jun 18;17(12):2029. doi: 10.3390/nu17122029 (PMC12196275; doi:10.3390/nu17122029)
Supplement: Supplementary file 1 [file nutrients-17-02029-s001.zip › nutrients-3684651-supplementary.pdf]

Online Supplementary Materials

METHODS.. 3

Method S1 Assessment for the AHA diet score.. 3

Method S2 Assessment for the AMED score 3

Method S3 Assessment for the AHEI score.. 4

Method S4 Assessment for the DASH score 4

Method S5 Assessment for the EAT-Lancet score.. 5

Method S6 Assessment for the MIND score 5

Method S7 Assessment for the Overall PDI, Healthful PDI, and Unhealthful PDI score. 6

TABLES 8

Table S1 Definition of Food groups, involving food items, Filed-ID of UK Biobank and corresponding CoFID codes of Composition of Foods Integrated Dataset 2021 from McCance and Widdowson's used in this study based on 24-hour recall. 8

Table S2 Nutrient, alcohol and energy intake metrics used in the present study............ 19

Table S3 Components and scoring criteria for the multiple dietary pattern scores: AHA score, aHEI score, MIND score, WCRF/AICR score, EAT-Lancet diet, AMED score, DASH score, DRRD score, and PDI scores (including oPDI hPDI, uPDI). 21

Table S4 Codes of disease used to identify prevalent comorbidities, translation pattern, and outcomes. 26

Table S5 Demographic characteristics at baseline (N = 206,463). 27

Table S6 Demographic characteristics at baseline across quintiles of AHA diet, AMED, and AHEI score (Quintile 1 vs. Quintile 5, N = 206,463). 29

Table S7 Demographic characteristics at baseline across quintiles of DASH, MIND, and EAT-Lancet score (Quintile 1 vs. Quintile 5, N = 206,463). 32

Table S8 Demographic characteristics at baseline across quintiles of Overall, Healthful, and Unhealthful PDI score (Quintile 1 vs. Quintile 5, N = 206,463). 35

Table S9 Hazard ratios (HR) and 95% Confidence Intervals (CI) for dietary pattern scores in relation to the risk of COPD-cause mortality. 38

Table S10 Net reclassification improvement (NRI), integrated discrimination improvement (IDI), and area under the ROC curve (AUC) for the risk of Chronic obstructive pulmonary disease (COPD) associated with multiple dietary patterns......... 41

Table S11 Hazard ratios (HR) and 95% confidence intervals (CI) for the risk of Chronic obstructive pulmonary disease (COPD) with multiple dietary patterns (Z-scores) from sensitivity analyses. 42

Table S12 Hazard ratios (HR) and 95% confidence intervals (CI) for stratified analyses and modified effects, with interaction assessed on a multiplicative scale, examining the association for the risk of COPD with Z-scores of AHA diet, AMED, AHEI-2010, DASH, or EAT-Lancet score. 43

Table S13 Hazard ratios (HR) and 95% confidence intervals (CI) for stratified analyses and modified effects, with interaction assessed on a multiplicative scale, examining the association for the risk of COPD with Z-scores of MIND, Overall PDI, Healthful PDI, or Unhealthful PDI score. 45

FIGURES 47

Figure S1 Dietary factors of dietary patterns.. 47

Figure S2 Potential non-linear relationship between multiple dietary patterns scores and the risk of COPD-caused mortality using restricted cubic spline regression. 49

REFERENCES 50

METHODS

Method S1 Assessment for the AHA diet score

The American Heart Association (AHA) diet score was developed based on Rehm's definition (1). This dietary pattern primarily emphasizes actionable, evidence-based priorities designed to enhance cardiometabolic health, as outlined in the AHA 2020 Strategic Impact Goals (2). The AHA diet score was based on the total consumption of eight dietary components, as outlined in e-Tables 1 and 2: (1) whole grains (1.1–1.9), (2) fruits and vegetables (12.1–12.6, 9.1–9.7), (3) nuts, seeds, and legumes (13.1–13.2), (4) sugar-sweetened beverages (SSBs) and fruit juices (16.1, 16.4), (5) processed meat (6.6), (6) fish and shellfish (7.1–7.5), (7) saturated fatty acids (N3), and (8) sodium (N14). As detailed in Table S3, each dietary component was assigned a score ranging from 0 to 10 based on whether its consumption was encouraged or discouraged. A score of 10 was given for optimal intake, defined as meeting or exceeding the AHA target for encouraged foods/nutrients, while a score of 0 was assigned for intakes below the target. For discouraged foods/nutrients, the scoring was reversed: a score of 10 was given for intakes at or below the AHA target, and a score of 0 for intakes exceeding the target. Intermediate intakes were scored proportionally, ranging from 1 to 9 points for each dietary component. The AHA diet score was calculated by summing the scores of all dietary components, with the total score ranging from 0 (indicating nonadherence) to a maximum of 80 (indicating perfect adherence).

Method S2 Assessment for the AMED score

The Mediterranean Diet (MED) is widely regarded as a healthy dietary pattern that helps prevent chronic diseases. The alternative Mediterranean Diet (AMED) is being investigated as a modified version to improve cultural adaptability (3). It retains the core elements of the MED while incorporating a diverse range of dietary components, including various food groups and nutrients, as detailed in e-Tables 1 and 2. Key dietary components emphasized in the AMED include: (1) whole grains (1.1–1.9), (2) fruits (12.1–12.6), (3) vegetables (excluding potatoes) (9.1–9.7), (4) nuts and seeds (13.1–13.2), (5) legumes (3.9, 8.2, 10.1–10.2), (6) fish and shellfish (7.1–7.3, 7.5), and (7) the MUFA to SFA ratio (N19). As outlined in Table S3, participants with intake above the median for these components received 1 point, while those below the median received 0 points. The restricted dietary component was (8) red and processed meat (6.2–6.4, 6.6), where intake below the median earned 1 point. Additionally,

participants were assigned 1 point for (9) alcohol intake (coded as N13), ranging from 10 to 25 g/day for males and 5 to 15 g/day for females. The AMED score ranged from 0 to 9, with higher scores reflecting greater adherence to the AMED pattern.

Method S3 Assessment for the AHEI score

The Alternate Healthy Eating Index (AHEI)-2010 was developed through a comprehensive review of relevant literature and consultations with nutrition researchers, building upon the original AHEI-2005 (4). The eleven dietary components of the AHEI-2010 are detailed in e-Tables 1 and 2, with the corresponding scoring criteria provided in Table S3. The scoring system of the AHEI-2010 includes six components for which higher intakes are considered optimal: (1) whole grains (1.1–1.9), (2) fruits (12.1–12.6), (3) vegetables (9.1–9.7), (4) nuts, seeds, and legumes (13.1–13.2, 3.9, 8.2, 10.1–10.2), (5) long-chain omega-3 fats, including eicosapentaenoic acid (EPA) and docosahexaenoic acid (DHA) (N22), and (6) polyunsaturated fatty acids (PUFA) excluding EPA and DHA (N23). One component, alcohol intake (N13), is considered optimal at a moderate level. Additionally, there are four components where lower intakes or avoidance are deemed optimal: (8) sugar-sweetened beverages (SSBs) and fruit juices (16.1, 16.4), (9) red and processed meats (6.2–6.4, 6.6), (10) trans-fatty acids (N18), and (11) sodium (N14, N24). To reduce recall bias, both estimated 24-hour sodium excretion and dietary sodium intake were assessed concurrently. Each dietary component was scored on a scale from 0 (worst) to 10 (best), with the total score ranging from 0 (indicating nonadherence) to 110 (indicating perfect adherence).

Method S4 Assessment for the DASH score

The DASH diet is widely endorsed by the National Heart, Lung, and Blood Institute for the prevention and management of hypertension (5). We developed a DASH score that reflects the consumption patterns recommended by the DASH diet, emphasizing or minimizing specific foods and nutrients. Detailed information is provided in e-Tables 1 and 2, which outline the constituent elements for each dietary component. This score is based on eight key dietary components. It promotes a high intake of (1) whole grains (1.1–1.9), (2) fruits and fruit juices (12.1–12.6, 16.1), (3) vegetables (9.1–9.7), (4) nuts, seeds, and legumes (13.1–13.2, 3.9, 8.2, 10.1–10.2), and (5) low-fat dairy products (N27). Concurrently, it advocates a low intake of (6) sugar-sweetened beverages (SSBs), excluding juices (16.4), (7) red and processed meats

(6.2–6.4, 6.6), and (8) sodium (N14, N24). To minimize recall bias, both estimated 24-hour sodium excretion and dietary sodium intake were considered together. For each dietary component, participants were categorized into quintiles based on their intake levels, with separate groupings for each sex. As detailed in Table S3, for components that were encouraged, the scoring system was structured such that the lowest quintile received 1 point and the highest quintile received 5 points. For components that were discouraged, the scoring system was reversed, with the highest quintile receiving 1 point and the lowest quintile receiving 5 points. The DASH score was then calculated by summing the individual component scores, yielding a total score range from 8 (indicating nonadherence) to 40 (indicating perfect adherence).

Method S5 Assessment for the EAT-Lancet score

In 2019, the EAT-Lancet Commission on Healthy Diets from Sustainable Food Systems established the first global reference diet (6). Subsequently, the details of the EAT-Lancet diet index were outlined in the development articles (7, 8). The EAT-Lancet diet comprises various dietary components, including food groups and nutrients (as detailed in e-Tables 1 and 2). For each component, target intake levels and reference intervals (ranges) are specified, with corresponding scores assigned in Table S3. The emphasized dietary components included: (1) whole grains (1.1–1.9), (2) fruits (12.1–12.6), (3) vegetables (9.1–9.7), (4) nuts and seeds (13.1–13.2), (5) legumes (3.9, 8.2, 10.1–10.2), (6) fish and shellfish (7.1–7.3, 7.5), and (7) the unsaturated fatty acids to saturated fatty acids (UFA:SFA) ratio (N21). The limited dietary components included: (8) potatoes (11.1–11.3), (9) beef and lamb (6.3–6.4), (10) pork (6.2), (11) poultry (6.1), (12) eggs (4.1), (13) dairy (N17), and (14) added sugar (N16). The boundaries for the different scores (0, 1, 2, or 3) were established based on the target intake levels and reference intervals. Each dietary component contributed between 0 and 3 points to the EAT-Lancet score, which ranged from 0 (indicating nonadherence) to 42 (indicating perfect adherence).

Method S6 Assessment for the MIND score

The Mediterranean-DASH Intervention for Neurodegenerative Delay (MIND) score is a hybrid of the Mediterranean and DASH diets, with a primary emphasis on dietary components and servings linked to neuroprotection and dementia prevention (9). In the present study, the MIND diet score includes 15 dietary components, comprising 10 healthy food groups: (1) whole grains (1.1–1.9), (2) berries (12.2), (3) green leafy

vegetables (9.2), (4) other vegetables (9.1, 9.3–9.7), (5) nuts and seeds (13.1–13.2), (6) legumes (3.9, 8.2, 10.1–10.2), (7) fish (7.1–7.3, 7.5), (8) poultry (6.1), (9) the monounsaturated to saturated fatty acid (MUFA:SFA) ratio (N19), as detailed in e-Tables 1 and 2. Due to limited information on the types of oils in the 24-hour dietary recall, the MUFA:SFA ratio was used as a dietary component, aligning with the Alternate Mediterranean Diet Score, rather than focusing specifically on olive oil consumption (3). One component for which moderate intake is considered ideal is (10) wine (17.1–17.3). The five unhealthy food groups are: (11) red and processed meats (6.2–6.4, 6.6), (12) butter and stick margarine (5.1, 5.2), (13) cheese (3.6, 3.7), (14) pastries and sweets (2.2, 14.1–14.8), and (15) fried/fast food (7.5, 9.7, 11.3). For all participants, the frequency of consumption for each dietary component was totaled, and a concordance score of 0, 0.5, or 1 was assigned. The overall MIND diet score was calculated by summing the scores for all 15 components, with a total score ranging from 0 (nonadherence) to 15 (perfect adherence). Details regarding the scoring are provided in Table S3.

Method S7 Assessment for the Overall PDI, Healthful PDI, and Unhealthful PDI score

Plant-based diets, often referred to as 'vegetarian' diets (10), categorize dietary components into three distinct groups: healthy plant foods, less healthy plant foods, and animal foods (11). The codes for the constituent elements of the plant-based diet are provided in e-Tables 1 and 2. The healthy plant foods category includes: (1) whole grains (1.1–1.9), (2) fruits (12.1–12.6), (3) vegetables (9.1–9.7), (4) nuts and seeds (13.1–13.2), (5) vegetarian protein sources (3.9, 8.2, 10.1–10.2, 8.1), and (6) tea and coffee (16.2–16.3). The less healthy plant foods category comprises: (7) refined grains (1.10–1.13), (8) potatoes (11.1–11.3), (9) fruit juices (16.1), (10) sugar-sweetened beverages (SSBs), excluding juice (16.4), and (11) sweets and desserts (2.2, 14.1–14.8). Lastly, the animal foods category includes: (12) meat (6.1–6.6), (13) fish or seafood (7.1–7.5), (14) eggs (4.1), (15) dairy (N17), and (16) animal fats (5.1, 5.2). Each dietary component, quantified in grams per day, was divided into sex-specific quintiles. As detailed in Table S3, for the overall PDI, positive scores were assigned to both healthy and less healthy plant food groups, while animal food groups received reverse scores. In contrast, when calculating the healthful PDI, positive scores were assigned only to the healthy plant food group, while reverse scores were given to both less healthy plant and animal food groups. The unhealthful PDI, on the other hand, awarded positive scores to the less healthy plant food group and reverse scores to both

the healthy plant and animal food groups. The scores from the 16 food groups were then aggregated for each individual to compute the overall, healthful, and unhealthful PDI. The total scores range from 16 points, indicating nonadherence, to 80 points, reflecting perfect adherence.

e-TABLES

Table S1 Definition of Food groups, involving food items, Filed-ID of UK Biobank and corresponding CoFID codes of Composition of Foods Integrated Dataset 2021 from McCance and Widdowson's used in this study based on 24-hour recall.

Components (Food groups)	Definition (involving food items)	Filed-ID of UK Biobank	Unit of questionnaire and definition portion size	ID of CoFID [a]
**1 Cereals & cereal products**				
1.1 Wholemeal bread	Wholemeal bread, sliced, baguette, bap, roll, couscous	20091/20092/20093/20094/ 100940/100101/100950/ 101020/101160/101230/ 101240/101270/102770	Slices/ items (1 Slice/ item =50 g)	11-981/11-982
1.2 Mixed bread	Mixed, brown or seeded bread, sliced, baguette, bap, roll			11-971/11-1136
1.3 Savory crackers	Oatcakes, crispbreads (including gluten free)	101250/101260	Items (1 item =10 g Crispbread or 15 g Oatcakes)	11-823/11-1100
1.4 Bran cereal	Bran cereal	100840	Bowls (1 bowl=40 g Bran cereal)	11-906
1.5 Biscuit cereal	Wholewheat biscuit cereal	100850	Bowls (1 bowl=40 g)	11-1034
1.6 Oat cereal (non-sugar)	Porridge oats (including milk/dried fruit added)	100770	Bowls (1 bowl=40 g)	11-789/11-788
1.7 Oat cereal (sugar)	Oatcrunch breakfast cereal	100810	Bowls (1 bowl=40 g)	11-795/11-793/11-757

1.8 Muesli	Muesli (with or without dried fruit)	100800	Bowls (1 bowl=60 g)	11-781/11-780
1.9 Wholemeal pasta, wholegrains	Brown and wholemeal pasta and rice	102720/102740/102780	Servings (1 serving = 50 g)	11-868/15-294
1.10 White bread	White bread, sliced, baguette, bap, roll	20091/20092/20093/20094/ 100940/100101/100950/ 101020/101160/101230/ 101240/101270	Slices/ items (1 Slice/ item =50 g)	11-980/11-1145
1.11 Other bread	Naan, garlic bread, other bread (including gluten free)			11-973/11-910
1.12 Other cereal (sugar)	Plain and sugary breakfast cereal (with/without dried fruit)	100820/100830/100860	Bowls (1 bowl=40 g)	11-1137/11-792
1.13 White pasta & rice	White pasta, rice, gluten free pasta	102710/102730	Servings (1 serving =70 g pasta or 50 g rice)	11-901/11-716/11-857
**2 Mixed dishes**				
2.1 Pizza	Pizza (including gluten free crust)	102000	Medium slices (1 medium slice = 200 g)	11-1016/11-936/11-1012/ 11-1011/11-1013/11-1015/ 11-1014/15-255
2.2 Grain dishes	Double and single crust pies, crumble pies, Yorkshire pudding, snackpot noodles	101970/101980/101990/ 102760	Servings/pots (1 serving/pot = 70 g) Slices/items (1 slices/item = 200 g)	11-1024/11-920/11-1026/ 19-559/11-1007/19-629/ 19-557/19-639/11-960/ 11-1146/11-921/18-505/

				18-506/11-1075/11-1135
2.3 Samosa, pakora	pakoras, onion bhajis, samosas	102040	Servings/pots (1 serving/pot = 40 g)	
2.4 Instant soups	Soups, powdered and canned	102520/102530/102540	Mugs/bowls (1 mug/bowl = 20 g dry weight)	17-850/17-653
2.5 Homemade Soups	Homemade Soups	102620		17-850/17-653
2.6 Sushi	Sushi	102750	Pieces (1 piece = 30 g)	16-361/16-362/15-617
**3 Dairy & dairy-free products**				
3.1 Whole milk	Whole milk >3.6 g fat per 100 g (cow, goat, sheep)	2104/2107		12-596/12-598/12-597/12-320
3.2 Semi-skimmed milk	Semi-skimmed milk >1 g fat per 100 g (cow, other)	2102	Glasses (1 glass = 240 ml or g)	12-313/12-418/12-419/12-314
3.3 Skimmed milk	Skimmed milk <1 g fat per 100 g (cow, cholesterol lowering, powdered)	2103		12-307/12-308/12-309/12-554
3.4 Full-fat yogurt	Whole milk yogurt (plain)	102080/ 102090/ 20106/	Servings (1 serving = 150 g)	12-375/12-530/12-184/12-515/12-531
3.5 Low-fat yogurt	Fat free and lower fat yogurt, plain			12-380/12-379/12-904/12-905"
3.6 High fat cheese	Cheese >17.5 g fat per 100 g, including hard cheese, soft cheese, spreadable, Blue, Feta, Mozzarella, Goats, other)	102820/102830/102840/102860/102870/102880/102890/102900/102910	Servings (1 serving = 30 g)	12-604/12-368/12-175
3.7 Medium & low-fat cheese	Cheese <=17.5g fat per 100 g, including hard and spreadable lower fat cheese, Cottage	102810/102850		12-550/12-538/12-541
3.8 Rice/oat drink	Rice and oat vegetable drinks	2108	Glasses (1 glass =	na

3.9 Soy drink	Soya drinks (including calcium fortified)	2105/2106	240 ml or g)	12-523/12-524
**4 Egg & egg dishes**				
**4.1 Eggs**	Whole eggs and processed (omelette, scotch eggs, other)	102940/102950/102960/ 102970/102980	Whole eggs (1 whole egg = 50 g)	12-941/12-938/12-937/ 12-815/12-816
**5 Fat & spreads**				
5.1 Olive oil (drizzling/dunking)	Olive oil	26110	g	17-038
5.2 Dairy fat spread lower fat	Spreadable/lower fat butter, dairy-based very low-fat spread			17-656
5.3 Dairy fat spread	Spreadable normal fat butter, dairy-based normal fat spread (including cholesterol lowering spread)	101300/101310/101350/ 101390/101430/101470/ 101510/101550/20098/ 20099/20100/20101/20102/ 20103/20104/20087	Thin/medium/thick (1 Thin/medium/thick = 1/1.5/2 g) 1 teaspoon = 5 g	17-655
5.4 Vegetable spread lower fat	Olive oil based lower fat spread, plant-based lower fat margarine and soya-based lower fat spread (including cholesterol lowering spread			12-498/17-710
5.5 Vegetable spread	Olive oil-based spread, plant-based soft or hard margarine and soya-based spread (including cholesterol lowering spread)			17-687/17-659/12-500/15-888
**6 Meat & meat products**				
6.1 Poultry	Poultry (with/without skin)	103060	Servings (1 serving = 150 g)	18-488/18-372/18-375/ 18-350
6.2 Pork	Pork	103030		18-559/18-600/18-608
6.3 Beef	Beef	103020		18-003/18-468
6.4 Lamb	Lamb	103040		18-478/18-098/18-475
6.5 Other meat, offal	Other meat including offal	103090	Servings (1 serving = 100 g)	18-402/18-404/18-406/ 18-409/18-413/18-

				415/18-417
6.6 Processed meat	Sausages, bacon (with and without fat), ham, liver pate	103010/103070/103080/ 103100	Sausages (1 sausage = 75 g)<br>Rashers (1 rasher =20 g)<br>Slices (1 slice =20 g)<br>Servings (1 serving = 100 g)	19-537/19-650/19-020/ 19-317/19-656/19-510/19-657
6.7 Breaded/battered Chicken	Fried poultry with batter/breadcrumbs	103050	Servings (1 serving = 100 g)	18-326/18-327/18-328/19-521
7.1 White fish & tinned tuna	Tinned tuna, white fish, Cod, Coley	103150/103190/103230		16-399/16-158
7.2 Oily fish	Oily fish, including salmon, Mackerel, Sardines, Herring, Bluefin, Trout, Eel, Kippers, Sprats	103160		16-175/16-397/16-393/ 16-360/16-401/16-224
7.3 Shellfish	shellfish, mussels, clams, oysters, winkles, and scallops/	103220		16-497/16-270/16-260/16-262
7.4 Prawns, lobster, crab	Prawns, lobster, crab, Langoustine	103200/103210	Servings (1 serving = 100 g)	16-391/16-452/16-441/ 16-470/16-392/16-332/ 16-343/16-388/16-481/ 16-387/16-482/16-384
7.5 Breaded/battered Fish	Fried fish with batter/breadcrumbs	103170/103180		16-427/16-426/16-428/ 16-444/16-445/16-052/ 16-053/16-055/16-127/ 16-128/16-138/16-139/ 16-140/16-219/16-220/

				16-164/16-165/16-166/ 16-167
**8 Meat substitutes**				
8.1 Vegetarian meals	Quorn-based and vegetarian burgers and products	103260/103280/103290	Servings (1 serving = 100 g)	13-574/15-720/15-839/ 15-262
8.2 Soy-based meals	Tofu-based products	103270		17-721
**9 Vegetables, excluding potatoes, Legumes/pulses, and Beans/peas**			1 cup vegetables = 236.59 g	
9.1 Raw salad	Mixed side salad, lettuce, watercress	104240/104370		13-520/15-871/13-669
9.2 Green leafy/cabbages	Broccoli, cabbage, kale, cauliflower, spinach, sprouts	104310/104300/104140/ 104180/104160	Servings (1 serving = 100 g)	13-502/13-177/13-582/ 13-512/13-234/13-572
9.3 Root vegetables	Beetroot, carrots, celery, parsnip, turnip	104360/104270/104190/ 104130/104170		13-164/13-496/13-448/ 13-312/13-389/13-636
9.4 Tomatoes	Fresh and tinned tomatoes	104340/104350	Medium fresh tomatoes （1 medium fresh tomato =150 g）	13-530/13-519
9.5 Allium vegetables	Garlic, leek, onion	104220/104230/104260	Cloves (1 clove = 1 g)	13-244/13-499/13-624
9.6 Other vegetables	Mushrooms, mixed vegetables, avocado, butternut squash, courgettes, peppers, sweetcorn	104380/104290/104200/ 104150/104210/104250/ 104060/104070/104100/104320	Servings (1 serving = 100 g)	13-505/13-318/13-609/ 15-621/14-386/13-355
9.7 Vegetable side dishes	Coleslaw, salad with added fat/mayonnaise	104080/ 104090		15-635/17-654
**10. Legumes and beans**				

10.1 Beans/peas	Broad bean, green bean, peas	104110/104120/104280	Servings (1 serving = 150 g)	13-608/13-438/13-064
10.2 Legumes/pulses	Baked beans, pulses	104000/104010		13-578/13-579
**11 Potatoes**				
11.1 Potatoes/sweet potatoes	Potatoes, sweet potatoes, boiled or baked	104330/104030	Servings (1 serving = 150 g)	13-618/13-489
11.2 Mashed potatoes	Potatoes, mashed	104050/		13-553
11.3 Fried/roast potatoes	Potatoes and chips, fried or roasted with fat	104020		13-599/13-600/13-492/13-534
**12. Fruits**			1 cup fruits = 236.59 g	
12.1 Citrus	Grapefruit, orange, satsuma	104490/104540/104530/	Servings (1 serving = 150 g)	14-327/14-384
12.2 Berries	Blackberries, strawberries, blueberries, raspberries, cherries	104470/104480	Servings (1 serving = 100 g)	14-324/14-388/14-382/14-375/14-325
12.3 Apples & pears	Apples and pears	104450/104560	Servings (1 serving = 150 g)	14-319/14-365
12.4 Bananas & other fruit	Bananas, mixed fruit, grapes, mango, melon, peach, pineapple, kiwi, other	104510/104520/104550/104570/104500/104460/104590/	Servings (1 serving = 150 g)	14-318/14-350/14-355/14-299/14-378/14-376
12.5 Dried fruit	Dried fruit, prunes	104430/104420	Servings (1 serving = 50 g)	14-231
12.6 Stewed fruit	Stewed fruit, plums	104580/104410/104440	Servings (1 serving = 150 g) Plums (1 plum =50 g)	14-316
**13 Nuts & seeds**				

13.1 Salted nuts & seeds	Salted peanuts and nuts	102410/102430	Handfuls (1 handful = 30 g)	14-834/15-221
13.2 Unsalted nuts & seeds	Unsalted peanuts and nuts	102420/102440/102450		14-877
**14 Sugar, preserves, cakes & confectionery, snacks**				
14.1 Added sugars & preserves	Table sugar, honey, jam and preserves	100370/ 100490/100900	Teaspoons (1 teaspoon = 4 g)	17-050/17-073/17-075/ 17-060/17-061/17-062/ 17-063
14.2 Chocolate confectionery	Chocolate bar (including white, milk and dark chocolate), chocolate-covered raisins, chocolate-covered sweets	102260/102270/102280/ 102290/102300/102310	Chocolate bars/handfuls (1 chocolate bar/handful =50 g)	17-650/17-690/17-648/ 17-491/17-091
14.3 Other sweets	Hard and soft sweets (including sugar free)	102380/102320/102330/ 100380/100500/100910	Items/handfuls (1 item/handful =50 g)	17-101/17-647/17-828
14.4 Savory snacks	Crisps, savory biscuits, cheese snacks, other savory biscuits	102460/102470/102480/ 102500	Bags/handfuls (1 bag/handful = 30 g)	11-821/11-807/11-808/ 11-801/11-797/17-665/ 17-773/17-775/12-540/ 12-537/11-824/11-952/ 11-1032
14.5 Biscuits	Chocolate biscuits, plain biscuits, sweet biscuits and cookies	102340/102350/102360	Items (1 item =30 g)	11-816/11-815/11-807/ 11-799/11-809/11- 810/11-811
14.6 Milk-dairy desserts	Ice cream, milk puddings, milk-based desserts, cheesecake	102120/102140/102150	Servings (1 serving = 100 g)	12-395/12-542/12-482/ 12-562/12-511/12-508

14.7 Desserts & cakes & pastries	Pancakes, croissant, Danish pastries, scones, fruitcakes, cakes, doughnuts, sponge puddings, other desserts, cereal bars, sweet snacks	102010/102050/102030/102060/102020/102070/102370/102180/102190		17-645/11-988/12-398/11-850/15-645/17-642/11-993/11-827/11-1041/11-1072/11-1125/11-849/11-959/11-1102/11-1103/15-833/11-1143/11-1141/11-1142/11-841/11-1119/11-1120/11-1121
14.8 Soya-based desserts & yogurt	Soya-based desserts	102170/102200/102210/102220/102230		15-668
**15. Sauces & condiments**				
15.1 Sauces	Yeast, chutney, olives, ketchup, brown sauce, tomato sauce	102490	Servings (1 serving = 50 g)	17-343/17-345/15-879/15-880/17-329/17-330/17-625/17-827/17-336/17-378/17-379/17-629/17-626/17-619/15-882
**16. Non-alcoholic beverages**				
16.1 Fruit juices	Orange, grapefruit drink and 100% fruit juice	100180/100190/100200/100210/100220	Glasses/cartons (1 glass/carton =250 ml or g)	17-845/17-737/17-744/17-735/17-848/17-674/17-740

16.2 Coffee	Normal instant, filter, cappuccino, espresso coffee/Decaffeinated instant, filter, cappuccino, espresso coffee	100250/100270/100290/ 100300/100310/100330	Cups/mugs (1 cup/mug =250 ml or g)	17-833/17-157/17-155/ 17-156/17-153/17-154/17-161
16.3 Tea	Black, green and other tea/Decaffeinated black, herbal tea, rooibos	100400/100410/100420/ 100430/100440		17-165/17-167/17-166/ 17-170/17-171/17-172/ 17-169/17-168
16.4 Sugar-sweetened beverages, excluding juice	Fizzy sugary drinks, squash, fruit smoothies, Low calorie fizzy drinks and squash	100170/100160	Glasses/cans (1 glass/can =250 ml or g)	17-672/17-747/13-643/ 13-357/17-742/17-846
16.5 Milk-based & powdered drinks	Dairy-based smoothies, milk-based drinks, hot chocolate	100230/100510/100530/ 100540/100550	Glasses/cartons (1 glass/carton =250 ml or 25g dry weight)	17-790/17-791/17-498/ 17-499/17-733/12-589/ 12-590/12-591
**17 Alcoholic beverages**			1 drink is 4 oz (113.4 ml or g) wine, 12 oz (340 ml or g) beer & cider, or 1.5 oz (42.5 ml or g) of Spirits	
17.1 White wine	White wine	100580/20095	Glasses (1 small/medium/large glass =125/175/250 ml or g)	17-756/17-755/17-233
17.2 Red wine	Red and rose wine	100590/20096/100630/ 20097		17-754/17-752
17.3 Fortified wine	Fortified wine	100720	Glasses (1 glass=75 ml or g)	17-234/17-236
17.4 Beer & Cider	Beer and cider	100710	Pints (1 pint= 586 ml	17-748/17-208/17-506

			or g)	
17.5 Spirits	Spirits and other alcoholic drinks	100730/100740	Measures (1 measures=25 ml or g)	17-247/17-758

[a] The UK Nutrient Databank food composition tables are openly accessible at www.gov.uk/government/publications/composition-of-foods-integrated-dataset-cofid; [b] The glycemic index for each food group was referenced from the website: https://glycemic-index.net/how-is-glycemic-index-calculated/. Abbreviations: CoFID, Composition of Foods Integrated Dataset.

Table S2 Nutrient, alcohol and energy intake metrics used in the present study.

No.	Nutrients and energy intake	Involving Food Groups/dietary related markers	Unit of calculation and definition portion size	Field-ID/Calculation Formula from CoFID
N1	Total energy intake	All food groups	KJ (1 KJ = 0.2389 kcal)	100002
N2	Carbohydrate	All food groups	g	26013
N3	SFA	All food groups	g	100006
N4	PUFA	All food groups	g	100007
N5	Toal sugars	All food groups	g	
N6	Natural sugars	9.1-9.7, 10.1-10.2, 12.1-12.6	g	
N7	Total fiber	All food groups	g	
N8	Cereal fiber	1.1-1.13	g	$= \sum_{1}^{N} (Foods\ intake\ (g) \times \frac{(Nutrients\ or\ Alcohol\ content\ per\ 100g\ of\ representive\ foods)}{100\ (g)})$
N9	Trans-fat acids	All food groups	g	
N10	MUFA	All food groups	g	
N11	EPA	All food groups	g	
N12	DHA	All food groups	g	
N13	Alcohol	17.7-17.5	g (1 serving = 10 g)	

N14	Sodium	All food groups	mg	
N16	Added Sugar	All food groups excluding 9.1-9.7, 10.1-10.2, 12.1-12.6	g	$= Total\ sugar - \ Natural\ sugar$
N17	Dairy [d]	3.1-3.9	g	$= Milk \times 1.0 + Cheese \times 5.0 + Cream \times 2.7 + Butter \times 6.5$
N18	Trans-fat acids	All food groups	% energy	$= \frac{\text{Trans} - \text{fat acids (g)} \times 9\ \text{(kcal/g)}}{\text{Total energy intake (kcal)}}$
N19	MUFA: SFA ratio	All food groups	--	$= \frac{\text{MUFA}}{\text{SFA}}$
N21	(MUFA+PUFA): SFA ratio	All food groups	--	$\frac{\text{MUFA} + \text{PUFA}}{\text{SFA}}$
N22	Long-chain (n-3) fats (EPA + DHA)	All food groups	mg	= EPA + DHA
N23	PUFA excluding EPA & DHA	All food groups	% energy	$\frac{(\text{PUFA} - \text{EPA} - \text{EPA})\ \text{(g)} \times 9\ \text{(kcal/g)}}{\text{Total energy intake (kcal)}}$
N24	Estimated 24-hour sodium excretion	Urinary biomarker data	mmol/L	30530
N27	Low fatty dairy (12)	3.2, 3.3, 3.5, 3.7	g	$= Milk\ (g) \times 1.0 + Cheese\ (g) \times 5.0 + Cream\ (g) \times 2.7 + Butter\ (g) \times 6.5$

Table S3 Components and scoring criteria for the multiple dietary pattern scores: AHA score, aHEI score, MIND score, WCRF/AICR score, EAT-Lancet diet, AMED score, DASH score, DRRD score, and PDI scores (including oPDI hPDI, uPDI).

Diet Pattern		Components [b]	Unit	Scoring criteria for dietary pattern for each component		
				**0 point**	**1–9 points [a]**	**10 points**
**AHA diet score**	1	Whole grain	oz/day	= 0	0–3.0	≥ 3.0
	2	Fruits and vegetables	cups/day	= 0	0–4.5	≥ 4.5
	3	Nuts, seeds and legumes	servings/day	= 0	0–1	≥ 4
	4	SSBs and fruit juice	fluid oz/day	≥ 16	16–5.1	<5.1
	5	Processed meat	oz/day	≥ 1.8	1.8–0.5	< 0.5
	6	Fish and shellfish	servings/week	= 0	0–2	≥ 2
	7	Saturated fat acids	% energy	≥ 15	15–7	≤ 7
	8	Sodium	g/day	≥ 4.5	4.5–1.5	≤ 1.5
				**0 point**	**1–9 points [a]**	**10 points**
**AHEI-2010 score**	1	Whole grains	g/day	0	M: 0–90<br>F: 0–75	M: ≥ 90<br>F: ≥ 75
	2	Fruits	servings/d	0	0–4	≥ 4
	3	Vegetables	servings/d	0	0–5	≥ 5
	4	Nuts, seeds and legumes	servings/d	0	0–1	≥ 1
	5	Long-chain (omega-3) fats	mg/d	0	0–250	≥ 250
	6	PUFA excluding EPA & DHA	% energy	≤ 2	2–10	≥ 10
	7	Alcohol	drinks/day	M: >3.5<br>F: >2.5	M: 3.5–2.0 or <0.5<br>F: 2.5–1.5 or <0.5	M: 0.5–2.0<br>F: 0.5–1.5
	8	SSBs and fruit juice	servings/d	≥ 1	1–0	0

	9	Red & processed meat	servings/d	≥ 1.5	1.5–0		0
	10	Trans-fat acids	% energy	≥ 4	4–0.5		≤ 0.5
	11	Sodium	mg/d<br>mmol/L	Sodium intake (6–0 points)<br>24-hour excretion (4–0 points)			
**MIND score**				**0 point**	**0.5 point**		**1 point**
	1	Whole grain	servings/day	<1	1–3		≥ 3
	2	Berries	serving/week	<1	1–2		≥ 2
	3	Green leafy vegetables	serving/week	<2	2–6		≥ 6
	4	Other vegetables, excluding Green leafy	serving/week	<5	5–7		≥ 7
	5	Nuts and seeds	serving/week	<0.23	0.23–5		≥ 5
	6	Legume	serving/week	<1	1–3		≥ 3
	7	Fish and shellfish	serving/week	< 0.23	0.23–1		≥ 1
	8	Poultry	serving/week	< 1	1–2		≥ 2
	9	MUFA: SFA ratio	--	< median	/		≥ median
	10	Wine	glass/week	≥ 7 or = 0	0–6		6–7
	11	Red & processed meat	serving/week	≥ 7	7–4		<4
	12	Butter and stick margarine	teaspoon/day	≥ 2	2–1		< 1
	13	Cheese	serving/week	≥ 7	7–1		< 1
	14	Pastries and sweets	serving/week	≥ 7	7–5		< 5
	15	Fried/fast food	serving/week	≥ 4	4–1		< 1
**EAT-Lancet diet**				**0 point**	**1 point**	**2 points**	**3 points**
	1	Whole grain	g/day	< 58	58–116	116–232	≥ 232
	2	Fruit	g/day	< 50	50–100	100–200	≥ 200
	3	Vegetables	g/day	< 100	100–200	200–300	≥ 300

	4	Nuts and seeds	g/day	< 12.5	12.5–25	25–50	≥ 50	
	5	Legume	g/day	< 18.75	18.75–37.5	37.5–75	≥ 75	
	6	Fish and shellfish	g/day	< 7	7–14	14–28	≥ 28	
	7	UFA: SFA ratio	na	< 0.4	0.4-0.6	0.6-0.8	≥ 0.8	
	8	Potatoes	g/day	≥ 200	100–200	50–100	< 50	
	9	Beef and lamb	g/day	≥ 28	14–28	7–14	< 7	
	10	Pork	g/day	≥ 28	14–28	7–14	< 7	
	11	Poultry	g/day	≥ 116	58-116	29-58	< 29	
	12	Eggs	g/day	≥ 50	25–50	13–25	< 13	
	13	Dairy	g/day	>1000	500–1000	250–500	< 250	
	14	Added sugar	g/day	≥ 124	62–124	31–62	< 31	
				**0 point**		**1 point**		
	1	Whole grains	g/day	< median		≥ median		
	2	Fruits	g/day	< median		≥ median		
	3	Vegetables	g/day	< median		≥ median		
	4	Nuts and seeds	g/day	< median		≥ median		
**AMED score**	5	Legumes	g/day	< median		≥ median		
	6	Fish and shellfish	g/day	< median		≥ median		
	7	MUFA: SFA ratio	na	< median		≥ median		
	8	Red & processed meat	g/day	≥ median		< median		
	9	Alcohol	g/day	M: <10 or >25<br>F: <5 or >15		M: 10–25<br>F: 5–15		
				**1 point**	**2 points**	**3 points**	**4 points**	**5 points**
	1	Whole grain	g/day	Quintile 1	Quintile 2	Quintile 3	Quintile 4	Quintile 5
**DASH score**	2	Fruit & Fruit juices	g/day	Quintile 1	Quintile 2	Quintile 3	Quintile 4	Quintile 5
	3	Vegetables	g/day	Quintile 1	Quintile 2	Quintile 3	Quintile 4	Quintile 5

	4	Nuts, seeds and legumes	g/day	Quintile 1	Quintile 2	Quintile 3	Quintile 4	Quintile 5
	5	Low fatty dairy	g/day	Quintile 1	Quintile 2	Quintile 3	Quintile 4	Quintile 5
	6	SSBs, excluding juice	g/day	Quintile 5	Quintile 4	Quintile 3	Quintile 2	Quintile 1
	7	Red & processed meat	g/day	Quintile 5	Quintile 4	Quintile 3	Quintile 2	Quintile 1
	8	Sodium	mg/day	Sodium intake (tertile 3–1, 3–1 points)				
			mmol/L	24-hour excretion (tertile 3–1, 2–0 points)				
**Plant-based diet score**				**1 point**	**2 points**	**3 points**	**4 points**	**5 points**
oPDI/ hPDI	1	Whole grain	g/day	Quintile 1	Quintile 2	Quintile 3	Quintile 4	Quintile 5
uPDI				Quintile 5	Quintile 4	Quintile 3	Quintile 2	Quintile 1
oPDI/ hPDI	2	Fruit	g/day	Quintile 1	Quintile 2	Quintile 3	Quintile 4	Quintile 5
uPDI				Quintile 5	Quintile 4	Quintile 3	Quintile 2	Quintile 1
oPDI/ hPDI	3	Vegetables	g/day	Quintile 1	Quintile 2	Quintile 3	Quintile 4	Quintile 5
uPDI				Quintile 5	Quintile 4	Quintile 3	Quintile 2	Quintile 1
oPDI/ hPDI	4	Nuts and seeds	g/day	Quintile 1	Quintile 2	Quintile 3	Quintile 4	Quintile 5
uPDI				Quintile 5	Quintile 4	Quintile 3	Quintile 2	Quintile 1
oPDI/ hPDI	5	Vegetarian protein resources	g/day	Quintile 1	Quintile 2	Quintile 3	Quintile 4	Quintile 5
uPDI				Quintile 5	Quintile 4	Quintile 3	Quintile 2	Quintile 1
oPDI/ hPDI	6	Tea and coffee	g/day	Quintile 1	Quintile 2	Quintile 3	Quintile 4	Quintile 5
uPDI				Quintile 5	Quintile 4	Quintile 3	Quintile 2	Quintile 1
oPDI/uPDI	7	Refined grains	g/day	Quintile 1	Quintile 2	Quintile 3	Quintile 4	Quintile 5
hPDI				Quintile 5	Quintile 4	Quintile 3	Quintile 2	Quintile 1
PDI/uPDI	8	Potatoes	g/day	Quintile 1	Quintile 2	Quintile 3	Quintile 4	Quintile 5
hPDI				Quintile 5	Quintile 4	Quintile 3	Quintile 2	Quintile 1
oPDI/uPDI	9	Fruit juices	g/day	Quintile 1	Quintile 2	Quintile 3	Quintile 4	Quintile 5
hPDI				Quintile 5	Quintile 4	Quintile 3	Quintile 2	Quintile 1
oPDI/uPDI	10	SSBs, excluding juice	g/day	Quintile 1	Quintile 2	Quintile 3	Quintile 4	Quintile 5

hPDI				Quintile 5	Quintile 4	Quintile 3	Quintile 2	Quintile 1
oPDI/uPDI	11	Sweets and desserts	g/day	Quintile 1	Quintile 2	Quintile 3	Quintile 4	Quintile 5
hPDI				Quintile 5	Quintile 4	Quintile 3	Quintile 2	Quintile 1
oPDI/uPDI/hPDI	12	Meat	g/day	Quintile 5	Quintile 4	Quintile 3	Quintile 2	Quintile 1
	13	Fish or seafood	g/day	Quintile 5	Quintile 4	Quintile 3	Quintile 2	Quintile 1
	14	Eggs	g/day	Quintile 5	Quintile 4	Quintile 3	Quintile 2	Quintile 1
	15	Dairy	g/day	Quintile 5	Quintile 4	Quintile 3	Quintile 2	Quintile 1
	16	Animal fat	g/day	Quintile 5	Quintile 4	Quintile 3	Quintile 2	Quintile 1

[a] Scored proportionally. [b] All dietary variables were energy adjusted to 2000 kcal/day using the residual method prior to analysis. All categories (median, tertiles, quartiles, quintiles) were divided according to sex-specific distribution. Abbreviations: AHA score, American Heart Association diet score; AHEI-2010 score, Alternate Healthy Eating Index; AMED score: alternate Mediterranean diet score; DASH score, Dietary Approaches to Stop Hypertension score; DHA, docosahexaenoic acid; EPA, eicosapentaenoic acid; MIND score, Mediterranean Dietary Approaches to Stop Hypertension Intervention for Neurodegenerative Delay diet score; MUFA, monounsaturated fatty acids; oPDI/hPDI/uPDI score, overall/healthful/unhealthful plant-based diet score; PUFA, polyunsaturated fatty acids; UFA, unsaturated fatty acids; SSB, sugar-sweetened beverages; SFA, saturated fatty acid; UFA , unsaturated fatty acids.

Table S4 Codes of disease used to identify prevalent comorbidities, and outcomes.

	Self-reported	ICD-9	ICD-10	
	Diagnoses	Diagnoses	Diagnoses	Death register: primary and secondary causes
Field ID	20002/ 87	41281/41271	41280/41270	40000/40001/40002
COPD	1112	4912, 496	J44	
CVD	1066, 1074, 1075, 1076, 1079, 1081, 1082, 1583, 1083, 1086	411, 412, 413, 414, 402, 404, 425, 428, 430, 431, 432, 433, 434, 435, 436, 437, 438	I20, I21, I22, I23, I24, I25, I11, I13, I42, I43, I50, I60, I61, I62, I63, I64, I65, I66, I67, I68, I69	
T2DM	1223	25000, 25010, 25020, 25030, 25040, 25050, 25060, 25070, 25080, 25090, 25002, 25012, 25022, 25032, 25042, 25052, 25062, 25072, 25082, 25092	E11, E12, E13, E14	
Hypertension	1065, 1072	401, 402, 403, 404, 405	I10, I11, I12, I13, I15, I674	
Other Respiratory diseases: e.g., asthma, emphysema, cystic fibrosis, pulmonary tuberculosis	1111, 1121, 1472, 1440	2770, 4160, 493, 492, 011	J45, J46, J43, A15, A16, E840, I270, I272	
The COPD-caused mortality	na	na	J44	

Abbreviations: CVD, cardiovascular disease; ICD, International Classification of Diseases; COPD, Chronic obstructive pulmonary disease; T2DM, type 2 diabetes mellitus.

Table S5 Demographic characteristics at baseline (N = 206,463).

Case/Total	Females<br>1071/113872	Males<br>1379/92591	Total<br>2450/206463
Age (years)	56 (49, 62)	58 (50, 63)	57 (50, 63)
White race (%)	108728 (95.48)	88518 (95.60)	197246 (95.54)
BMI (kg/m^2)	25.6 (23.1, 29.0)	26.9 (24.7, 29.6)	26.2 (23.7, 29.3)
Smoking status (%)			
Never	69186 (60.76)	47680 (51.50)	116866 (56.60)
Previous	36896 (32.40)	36204 (39.10)	73100 (35.41)
Only occasionally	2130 (1.87)	2839 (3.07)	4969 (2.41)
Almost all days	5373 (4.72)	5614 (6.06)	10987 (5.32)
Missing data	287 (0.25)	254 (0.27)	541 (0.26)
FEV1/FVC ratio	0.77 (0.74, 0.80)	0.76 (0.72, 0.80)	0.77 (0.73, 0.80)
FEV1 (L)	2.49 (2.17, 2.82)	3.42 (2.97, 3.87)	2.83 (2.38, 2.41)
FVC (L)	3.35 (2.86, 3.65)	4.51 (3.98, 5.08)	3.73 (3.14, 4.48)
Missing data (%)	29919 (26.27)	22573 (24.38)	52492 (25.42)
Alcohol consumption (%)			
Never	4736 (4.16)	1873 (2.02)	6609 (3.20)
Previous	3492 (4.16)	2684 (2.90)	6176 (2.99)
Current	105531 (92.68)	87954 (94.99)	193485 (93.71)
Missing data (%)	113 (0.10)	80 (0.09)	193 (0.09)
Physical activity (%)			
Low	16688 (14.66)	15506 (16.75)	32194 (15.59)
Moderate	41225 (36.20)	32981 (35.62)	74206 (35.94)
High	35504 (31.18)	33137 (35.79)	68641 (33.25)
Missing data (%)	20455 (17.96)	10967 (11.84)	31422 (15.22)
Total energy intake (kcal/day)	1903 (1599, 2235)	2215 (1854, 2619)	2030 (1694, 2418)
Townsend index	-2.28 (-3.70, 0.10)	-2.37 (-3.77, -0.01)	-2.32 (-3.73, -0.04)
Educational level (%)			
High	46985 (41.26)	41219 (44.52)	88204 (42.72)
Moderate	41755 (36.67)	27849 (30.08)	69604 (33.71)
Low	25132 (22.07)	23523 (25.41)	48655 (23.57)
CVD (%)	3678 (3.23)	7522 (8.12)	11200 (5.42)
T2DM (%)	2932 (2.57)	4734 (5.11)	7666 (3.71)
Hypertension (%)	50815 (44.62)	55610 (60.06)	106425 (51.55)
Respiratory diseases (%)	13869 (12.18)	10217 (11.03)	24086 (11.67)
Occupation-related breathing problems (%)	32420 (28.47)	30926 (33.40)	63346 (30.68)
Air pollution (mg/m^3)			
NO	41.8 (33.8, 50.1)	41.5 (33.5, 49.8)	41.7 (33.7, 49.9)

NO_2	26.0 (21.1, 31.3)	25.8 (21.0, 31.1)	25.9 (21.0, 31.2)
NO_X	67.9 (55.2, 81.0)	67.4 (54.9, 80.5)	67.7 (55.1, 80.8)
PM: 2.5 μm	9.87 (9.23, 10.5)	9.86 (9.22, 10.5)	9.87 (9.23, 10.5)
PM: 2.5-10 μm	6.12 (5.84, 6.63)	6.12 (5.84, 6.63)	6.12 (5.84, 6.63)
PM: 10 μm	16.1 (15.2, 17.0)	16.0 (15.2, 17.0)	16.1 (15.2, 17.0)

Continuous variables are presented as medians with interquartile ranges, and categorical variables are expressed as numbers with corresponding percentages. Abbreviations: BMI, body mass index; CVD, cardiovascular disease; PM, Particulate matter; T2DM, type 2 diabetes mellitus; NO, Nitrogen oxides; NO_2, Nitrogen dioxide.

Table S6 Demographic characteristics at baseline across quintiles of AHA diet, AMED, and AHEI score (Quintile 1 vs. Quintile 5, N = 206,463).

	AHA diet score		AMED score		AHEI-2010 score	
	Quintile 1 (0–33 points)	Quintile 5 (54–80 points)	Quintile 1 (0–2 points)	Quintile 5 (6–45 points)	Quintile 1 (-2085–22 points)	Quintile 5 (119–2909 points)
Case/Total	669/42307	375/42655	565/36727	655/40483	756/41289	403/41289
Age (years)	55 (48, 61)	59 (52, 63)	55 (48, 61)	55 (47, 61)	54 (47, 61)	59 (52, 63)
White race (%)	40289 (95.23)	40809 (95.67)	35133 (95.66)	38779 (95.79)	39229 (95.01)	39101 (94.70)
Male (%)	18894 (44.66)	19148 (44.89)	16077 (43.77)	18102 (44.72)	18517 (44.85)	18517 (44.85)
BMI (kg/m^2)	27.1 (24.4, 30.4)	25.5 (23.2, 28.2)	26.8 (24.1, 30.0)	27.1 (24.4, 30.4)	29.1 (24.2, 30.2)	26.2 (23.7, 29.3)
Smoking status (%)						
Never	23531 (55.62)	24585 (57.64)	19996 (54.44)	21194 (52.35)	22451 (54.38)	23466 (56.83)
Previous	14144 (33.43)	15729 (36.87)	12461 (33.93)	14293 (35.31)	13395 (32.44)	15529 (37.61)
Only occasionally	1113 (2.63)	928 (2.18)	947 (2.58)	1199 (2.96)	1135 (2.75)	945 (2.29)
Almost all days	3363 (7.95)	1316 (3.09)	3226 (8.78)	3670 (9.07)	4189 (10.15)	1244 (3.01)
Missing data	156 (0.37)	97 (0.23)	97 (0.26)	127 (0.31)	119 (0.29)	105 (0.25)
FEV1/FVC ratio	0.77 (0.73, 0.80)	0.76 (0.73, 0.80)	0.77 (0.73, 0.80)	0.77 (0.73, 0.80)	0.77 (0.73, 0.80)	0.77 (0.73, 0.80)
FEV1 (L)	2.82 (2.37, 3.39)	2.85 (2.39, 3.43)	2.84 (2.37, 3.42)	2.84 (2.38, 3.41)	2.82 (2.36, 3.39)	2.82 (2.36, 3.39)
FVC (L)	3.69 (3.11, 4.43)	3.75 (3.16, 4.53)	3.73 (3.14, 4.49)	3.72 (3.13, 4.46)	3.71 (3.13, 4.45)	3.71 (3.12, 4.46)
Missing data (%)	11123 (26.30)	10613 (24.88)	9567 (26.05)	10261 (25.35)	11093 (26.87)	10706 (25.93)
Alcohol consumption (%)						
Never	1560 (3.69)	1164 (2.73)	1226 (3.34)	1099 (2.71)	1582 (3.83)	1518 (3.68)
Previous	1527 (3.61)	1124 (2.64)	1140 (3.10)	1004 (2.48)	1381 (3.34)	1548 (3.75)

Current	39171 (92.59)	40334 (94.56)	34317 (93.44)	38334 (94.69)	38277 (92.71)	38186 (92.48)
Missing data	49 (0.12)	33 (0.08)	44 (0.12)	46 (0.11)	49 (0.12)	37 (0.09)
Physical activity (%)						
Low	8161 (19.29)	4942 (11.59)	6899 (18.78)	7867 (19.43)	8339 (20.20)	4921 (11.92)
Moderate	14835 (35.07)	15273 (35.81)	12946 (35.25)	14395 (35.56)	14240 (34.49)	14193 (34.37)
High	12227 (28.90)	16568 (38.84)	10716 (29.18)	11553 (28.54)	11443 (27.71)	16410 (39.74)
Missing data	7084 (16.74)	5872 (13.77)	6166 (16.79)	6668 (16.47)	7267 (17.60)	5765 (13.96)
Total energy intake (kcal/day)	1762 (1475, 2074)	2261 (1927, 2637)	2301 (2003, 2685)	1950 (1625, 2333)	1947 (1592, 2352)	1830 (1520, 2180)
Townsend index	-2.18 (-3.65, 0.37)	-2.41 (-3.80, -0.09)	-2.16 (-3.63, 0.31)	-2.17 (-3.64, 0.34)	-2.07 (-3.58, 0.55)	-2.36 (-3.76, -0.00)
Educational level (%)						
High	15107 (35.71)	20811 (48.79)	13251 (36.08)	14608 (36.08)	13489 (32.67)	18837 (45.62)
Moderate	15449 (36.52)	13254 (31.07)	13612 (37.06)	14976 (36.99)	15426 (37.36)	13110 (31.75)
Low	11751 (27.78)	8590 (20.14)	9864 (26.86)	10899 (26.92)	12374 (29.97)	9342 (22.63)
CVD (%)	2496 (5.90)	2206 (5.17)	1840 (5.01)	2185 (5.40)	2319 (5.60)	2527 (6.12)
T2DM (%)	1929 (4.56)	1349 (3.16)	1251 (3.41)	1558 (3.85)	1683 (4.08)	1742 (4.22)
Hypertension (%)	22042 (52.10)	22026 (51.64)	18450 (50.24)	21272 (52.55)	21286 (51.55)	22295 (54.00)
Respiratory diseases (%)	5219 (12.34)	4708 (11.04)	4535 (12.35)	5010 (12.38)	4990 (12.09)	4844 (11.73)
Occupation-related breathing problems (%)	11710 (27.68)	14069 (32.98)	10181 (27.72)	11526 (28.47)	11252 (27.25)	12172 (29.48)
Air pollution (mg/m^3)						
NO	42.1 (34.2, 50.0)	41.4 (33.4, 50.1)	42.2 (34.1, 50.1)	41.6 (33.3, 50.1)	42.1 (34.1, 50.2)	41.6 (33.5, 50.1)
NO_2	26.0 (21.3, 31.1)	26.0 (20.9, 31.4)	26.1 (21.3, 31.2)	25.9 (20.9, 31.4)	26.0 (21.3, 31.2)	26.0 (21.0, 31.4)
NO_X	68.1 (55.9, 80.7)	67.6 (54.7, 81.2)	68.2 (55.9, 81.0)	67.6 (54.5, 81.2)	68.2 (55.8, 81.0)	67.7 (54.8, 81.1)

PM 2.5 μm	9.81 (9.08, 10.4)	9.78 (9.06, 10.4)	9.90 (9.27, 10.5)	9.86 (9.21, 10.5)	9.90 (9.27, 10.5)	9.86 (9.21, 10.5)
PM 2.5-10 μm	6.13 (5.85, 6.65)	6.12 (5.84, 6.62)	6.12 (5.85, 6.64)	6.12 (5.84, 6.64)	6.13 (5.85, 6.66)	6.11 (5.84, 6.62)
PM 10 μm	16.1 (15.3, 17.0)	16.0 (15.2, 17.0)	16.1 (15.3, 17.0)	16.1 (15.2, 17.0)	16.1 (15.3, 17.1)	16.1 (15.2, 17.0)

Continuous variables are presented as medians with interquartile ranges, and categorical variables are expressed as numbers with corresponding percentages. Abbreviations: BMI, body mass index; CVD, cardiovascular disease; PM, Particulate matter; T2DM, type 2 diabetes mellitus; NO, Nitrogen oxides; NO_2, Nitrogen dioxide.

Table S7 Demographic characteristics at baseline across quintiles of DASH, MIND, and EAT-Lancet score (Quintile 1 vs. Quintile 5, N = 206,463).

	DASH score		MIND score		EAT-Lancet score	
	Quintile 1 (3–20 points)	Quintile 5 (3–20 points)	Quintile 1 (0–4.5 points)	Quintile 5 (9–22 points)	Quintile 1 (0–2.5 points)	Quintile 5 (4.25–6 points)
Case/Total	755/46191	755/46191	700/40525	712/44399	528/38843	466/42600
Age (years)	54 (47, 61)	54 (47, 61)	55 (48, 62)	55 (48, 62)	56 (48, 62)	58 (51, 63)
White race (%)	43749 (94.71)	43749 (94.71)	38808 (95.76)	42424 (95.55)	37800 (97.31)	39414 (92.52)
Male (%)	20832 (45.10)	20832 (45.10)	17826 (43.99)	19140 (43.11)	15688 (40.39)	18804 (44.14)
BMI (kg/m^2)	27.2 (24.5, 30.6)	27.2 (24.5, 30.6)	26.9 (24.2, 30.2)	27.0 (24.3, 30.2)	26.6 (24.0, 29.8)	25.9 (23.4, 28.9)
Smoking status (%)						
Never	24481 (53.00)	24481 (53.00)	21764 (53.71)	24300 (54.73)	21166 (54.49)	25233 (59.23)
Previous	15966 (34.57)	15966 (34.57)	13928 (34.37)	15115 (34.04)	13959 (35.94)	14570 (34.20)
Only occasionally	1330 (2.88)	1330 (2.88)	1075 (2.65)	1134 (2.55)	999 (2.57)	895 (2.10)
Almost all days	4275 (9.26)	4275 (9.26)	3637 (8.97)	3715 (8.37)	2595 (6.68)	1820 (4.27)
Missing data	139 (0.30)	139 (0.30)	121 (0.30)	135 (0.30)	124 (0.32)	82 (0.19)
FEV1/FVC ratio	0.77 (0.73, 0.80)	0.77 (0.73, 0.80)	0.77 (0.73, 0.80)	0.77 (0.73, 0.80)	0.77 (0.73, 0.80)	0.77 (0.73, 0.80)
FEV1 (L)	2.84 (2.38, 3.41)	2.84 (2.38, 3.41)	2.81 (2.35, 3.39)	2.80 (2.34, 3.37)	2.78 (2.35, 3.32)	2.84 (2.37, 3.43)
FVC (L)	3.73 (3.13, 4.47)	3.73 (3.13, 4.47)	3.70 (3.10, 4.45)	3.67 (3.09, 4.42)	3.64 (3.09, 4.35)	3.74 (3.14, 4.52)
Missing data (%)	12143 (26.29)	12143 (26.29)	10707 (26.42)	11478 (25.85)	9467 (24.37)	11915 (27.97)
Alcohol consumption (%)						
Never	1463 (3.17)	1463 (3.17)	1499 (3.70)	1532 (3.45)	582 (1.50)	2268 (5.32)
Previous	1429 (3.09)	1429 (3.09)	1440 (3.55)	1334 (3.00)	643 (1.66)	2025 (4.75)
Current	43248 (93.63)	43248 (93.63)	37543 (92.64)	41481 (93.43)	37580 (96.75)	38273 (89.84)

Missing data	51 (0.11)	51 (0.11)	43 (0.11)	52 (0.12)	38 (0.10)	34 (0.08)
Physical activity (%)						
Low	9151 (19.81)	9151 (19.81)	7495 (18.49)	8549 (19.25)	7013 (18.05)	5574 (13.08)
Moderate	16069 (34.79)	16069 (34.79)	14048 (34.67)	15491 (34.89)	14102 (36.31)	14726 (34.57)
High	13262 (28.71)	13262 (28.71)	11935 (29.45)	12838 (28.92)	11336 (29.18)	16047 (37.67)
Missing data	7709 (16.69)	7709 (16.69)	7047 (17.39)	7521 (16.94)	6392 (16.46)	6253 (14.68)
Total energy intake (kcal/day)	2070 (1702, 2489)	2070 (1702, 2489)	2078 (1718, 2509)	1958 (1626, 2342)	1841 (1576, 2134)	2176 (1782, 2596)
Townsend index	-2.06 (-3.60, 0.50)	-2.06 (-3.60, 0.50)	-2.10 (-3.61, 0.44)	-2.22 (-3.66, 0.25)	-2.35 (-3.74, 0.02)	-2.19 (-3.68, 0.23)
Educational level (%)						
High	16134 (34.93)	16134 (34.93)	13818 (34.10)	15010 (33.81)	15031 (38.70)	19196 (45.06)
Moderate	17227 (37.30)	17227 (37.30)	14805 (36.53)	16687 (37.58)	14219 (36.61)	13591 (31.90)
Low	12830 (27.78)	12830 (27.78)	11902 (29.37)	12702 (28.61)	9593 (24.70)	9813 (23.04)
CVD (%)	2447 (5.30)	2447 (5.30)	2311 (5.70)	2485 (5.60)	2138 (5.50)	2202 (5.17)
T2DM (%)	2015 (4.36)	2015 (4.36)	1659 (4.09)	1679 (3.78)	1400 (3.60)	1654 (3.88)
Hypertension (%)	23904 (51.75)	23904 (51.75)	20860 (51.47)	23220 (52.30)	20461 (52.68)	21256 (49.90)
Respiratory diseases (%)	5848 (12.66)	5848 (12.66)	4955 (12.23)	5411 (12.19)	4718 (12.15)	4802 (11.27)
Occupation-related breathing problems (%)	13362 (28.93)	13362 (28.93)	10400 (25.66)	13102 (29.51)	12470 (32.10)	11696 (27.46)
Air pollution (mg/m^3)						
NO	42.4 (34.5, 50.4)	41.4 (33.2, 49.9)	42.2 (34.1, 50.0)	41.6 (33.3, 50.3)	41.8 (34.0, 49.6)	42.2 (33.8, 51.2)
NO_2	26.3 (21.5, 31.4)	25.8 (20.8, 31.2)	26.1 (21.4, 31.1)	26.0 (20.9, 31.5)	25.8 (21.2, 30.9)	26.4 (21.1, 32.0)
NO_X	68.7 (56.5, 81.5)	67.4 (54.4, 80.7)	68.2 (56.2, 80.8)	67.7 (54.6, 81.5)	67.7 (55.6, 80.2)	68.7 (55.4, 82.9)
PM 2.5 µm	9.92 (9.30, 10.5)	9.84 (9.20, 10.5)	9.90 (9.29, 10.5)	9.86 (9.21, 10.5)	9.88 (9.26, 10.5)	9.89 (9.23, 10.5)

PM 2.5-10 μm	6.13 (5.85, 6.66)	6.11 (5.84, 6.62)	6.12 (5.85, 6.63)	6.12 (5.84, 6.65)	6.12 (5.85, 6.64)	6.13 (5.85, 6.64)
PM 10 μm	16.1 (15.3, 17.1)	16.0 (15.2, 17.0)	16.1 (15.3, 17.1)	16.0 (15.2, 17.0)	16.1 (15.2, 17.0)	16.1 (15.3, 17.1)

Continuous variables are presented as medians with interquartile ranges, and categorical variables are expressed as numbers with corresponding percentages. Abbreviations: BMI, body mass index; CVD, cardiovascular disease; PM, Particulate matter; T2DM, type 2 diabetes mellitus; NO, nitrogen oxides; NO_2, nitrogen dioxide.

Table S8 Demographic characteristics at baseline across quintiles of Overall, Healthful, and Unhealthful PDI score (Quintile 1 vs. Quintile 5, N = 206,463).

	Overall PDI score		Healthful PDI score		Unhealthful PDI score	
	Quintile 1 (3–46 points)	Quintile 5 (56–72 points)	Quintile 1 (3–45 points)	Quintile 5 (57–80 points)	Quintile 1 (3–45 points)	Quintile 5 (57–80 points)
Case/Total	522/41525	402/42150	574/41574	403/40723	408/40958	510/40615
Age (years)	56 (48, 62)	58 (51, 63)	55 (48, 61)	58 (51, 63)	59 (52, 63)	54 (47, 61)
White race (%)	39438 (94.97)	40371 (95.78)	39370 (94.70)	38973 (95.70)	39291 (95.93)	38583 (95.00)
Male (%)	19105 (46.01)	19442 (46.13)	18672 (44.91)	18373 (45.12)	19958 (48.73)	19698 (48.50)
BMI (kg/m^2)	26.7 (24.0, 29.8)	25.9 (23.5, 28.8)	27.1 (24.4, 30.3)	25.5 (23.2, 28.4)	26.2 (23.7, 29.1)	26.8 (24.2, 30.0)
Smoking status (%)						
Never	21249 (51.17)	25521 (60.55)	23584 (56.73)	23239 (57.07)	22268 (54.37)	23477 (57.80)
Previous	15551 (37.45)	14324 (33.98)	13892 (33.42)	14806 (36.36)	15868 (38.74)	13263 (32.66)
Only occasionally				925 (2.27)	1070 (2.61)	995 (2.45)
Almost all days	4617 (11.12)	2200 (5.22)	3976 (9.57)	1657 (4.07)	1660 (4.05)	2763 (6.80)
Missing data	108 (0.26)	105 (0.25)	122 (0.29)	96 (0.24)	92 (0.22)	117 (0.29)
FEV1/FVC ratio	0.77 (0.73, 0.80)	0.77 (0.73, 0.80)	0.77 (0.73, 0.80)	0.76 (0.73, 0.80)	0.77 (0.73, 0.80)	0.77 (0.73, 0.80)
FEV1 (L)	2.87 (2.40, 3.45)	2.85 (2.38, 3.41)	2.82 (2.37, 3.40)	2.86 (2.40, 3.45)	2.85 (2.38, 3.43)	2.91 (2.42, 3.50)
FVC (L)	3.76 (3.18, 4.54)	3.74 (3.14, 4.49)	3.69 (3.11, 4.44)	3.78 (3.17, 4.55)	3.75 (3.15, 4.50)	3.82 (3.19, 4.59)
Missing data (%)	10688 (25.74)	10813 (25.65)	10923 (26.27)	10207 (25.06)	10008 (24.43)	10878 (26.78)
Alcohol consumption (%)						
Never	931 (2.24)	1803 (4.28)	1514 (3.64)	1305 (3.20)	1073 (2.62)	1529 (3.76)
Previous	950 (2.29)	1668 (3.96)	1306 (3.64)	1350 (3.32)	1279 (3.12)	1297 (3.19)
Current	39601 (95.37)	38653 (91.70)	38712 (93.12)	38041 (93.41)	38571 (94.17)	37743 (92.93)

Missing data	43 (0.10)	26 (0.06)	42 (0.10)	27 (0.07)	35 (0.09)	46 (0.11)
Physical activity (%)						
Low	7198 (17.33)	5794 (13.75)	8265 (19.88)	4698 (11.54)	5410 (13.21)	7339 (18.07)
Moderate	14788 (35.61)	15151 (35.95)	14780 (35.55)	14434 (35.44)	14737 (35.98)	14326 (35.27)
High	13274 (31.97)	14931 (35.42)	11652 (28.03)	15809 (38.82)	15224 (37.17)	12275 (30.22)
Missing data	6265 (15.09)	6274 (14.88)	6877 (16.54)	5782 (14.20)	5587 (13.64)	6675 (16.43)
Total energy intake (kcal/day)	2258 (1878, 2682)	1921 (1602, 2255)	1794 (1504, 2122)	2296 (1986, 2669)	1958 (1626, 2342)	2385 (2072, 2742)
Townsend index	-2.02 (-3.60, 0.53)	-2.45 (-3.79, -0.24)	-2.19 (-3.65, 0.32)	-2.36 (-3.75, 0.01)	-2.35 (-3.76, 0.00)	-2.21 (-3.65, 0.25)
Educational level (%)						
High	17513 (42.17)	18436 (43.74)	15406 (37.06)	19260 (47.30)	19349 (47.24)	14778 (36.39)
Moderate	14134 (34.04)	13834 (32.82)	15271 (36.73)	12803 (31.44)	12653 (30.89)	15023 (36.99)
Low	9878 (23.79)	9880 (23.44)	10897 (26.21)	8660 (21.27)	8956 (21.87)	10814 (26.63)
CVD (%)	2001 (4.82)	2705 (6.42)	2421 (5.82)	2112 (5.19)	2433 (5.94)	2197 (5.41)
T2DM (%)	1437 (3.46)	1694 (4.02)	1656 (3.98)	1438 (3.53)	1818 (4.44)	1453 (3.58)
Hypertension (%)	21047 (50.69)	22224 (52.73)	21755 (52.33)	20509 (50.36)	21955 (53.60)	20844 (51.32)
Respiratory diseases (%)	4844 (11.67)	4921 (11.67)	5116 (12.31)	4581 (11.25)	4497 (10.98)	5108 (12.58)
Occupation-related breathing problems (%)	11982 (28.85)	13370 (31.72)	13011 (31.30)	11585 (28.45)	13129 (32.05)	11388 (28.04)
Air pollution (mg/m^3)						
NO	42.6 (34.4, 51.2)	41.1 (33.3, 49.2)	42.1 (34.3, 50.2)	41.7 (33.5, 50.2)	41.5 (33.1, 50.2)	42.0 (34.3, 49.8)
NO_2	26.6 (21.5, 31.9)	25.5 (20.8, 30.7)	26.1 (21.4, 31.2)	26.0 (21.0, 31.4)	25.9 (20.8, 31.3)	26.0 (21.4, 31.1)
NO_X	69.3 (56.3, 82.8)	66.7 (54.5, 79.6)	68.2 (56.1, 81.2)	67.8 (54.8, 81.3)	67.5 (54.3, 81.1)	68.1 (56.1, 80.6)
PM: 2.5 μm	9.84 (9.09, 10.5)	9.77 (9.07, 10.4)	9.90 (9.27, 10.5)	9.86 (9.22, 10.5)	9.79 (9.05, 10.4)	9.81 (9.10, 10.4)
PM: 2.5-10 μm	6.15 (5.86, 6.67)	6.10 (5.84, 6.59)	6.14 (5.86, 6.66)	6.11 (5.84, 6.60)	6.12 (5.84, 6.64)	6.12 (5.85, 6.12)

PM: 10 μm	16.1 (15.3, 17.1)	16.0 (15.2, 16.9)	16.1 (15.3, 17.1)	16.0 (15.2, 17.0)	16.0 (15.2, 17.0)	16.1 (15.3, 17.0)

Continuous variables are presented as medians with interquartile ranges, and categorical variables are expressed as numbers with corresponding percentages. Abbreviations: BMI, body mass index; CVD, cardiovascular disease; PM, Particulate matter; T2DM, type 2 diabetes mellitus; NO, nitrogen oxides; NO_2, nitrogen dioxide.

Table S9 Hazard ratios (HR) and 95% Confidence Intervals (CI) for dietary pattern scores in relation to the risk of COPD-cause mortality.

	Case/N	Model 1		Model 2		Model 3	
		HR (95%CI)	*P* (trend) value	HR (95%CI)	*P* (trend) value	HR (95%CI)	*P* (trend) value
AHA diet score							
Quintile 1	83/42307	1 (Ref.)		1 (Ref.)		1 (Ref.)	
Quintile 2	59/42388	0.71 (0.51, 0.99)		0.63 (0.45, 0.89)		0.80 (0.57, 1.12)	
Quintile 3	39/38401	0.52 (0.35, 0.75)	<0.001	0.43 (0.29, 0.63)	<0.001	0.65 (0.44, 0.97)	0.005
Quintile 4	35/40712	0.44 (0.29, 0.65)		0.35 (0.24, 0.52)		0.58 (0.38, 0.87)	
Quintile 5	38/42655	0.45 (0.31, 0.66)		0.33 (0.23, 0.49)		0.61 (0.40, 0.93)	
Z-score	254/206463	0.72 (0.64, 0.81)	<0.001	0.63 (0.56, 0.71)	<0.001	0.80 (0.70, 0.91)	0.001
AMED score							
Quintile 1	70/36727	1 (Ref.)		1 (Ref.)		1 (Ref.)	
Quintile 2	45/36541	0.64 (0.44, 0.94)		0.58 (0.40, 0.84)		0.67 (0.46, 0.98)	
Quintile 3	51/42919	0.62 (0.43, 0.89)	<0.001	0.52 (0.36, 0.74)	<0.001	0.61 (0.42, 0.90)	<0.001
Quintile 4	53/40233	0.69 (0.48, 0.98)		0.55 (0.38, 0.78)		0.70 (0.47, 1.02)	
Quintile 5	35/50043	0.37 (0.24, 0.55)		0.27 (0.18, 0.41)		0.37 (0.24, 0.58)	
Z-score	254/206463	0.73 (0.65, 0.83)	<0.001	0.65 (0.58, 0.74)	<0.001	0.73 (0.65, 0.83)	<0.001
AHEI-2010 score							
Quintile 1	88/40483	1 (Ref.)		1 (Ref.)		1 (Ref.)	
Quintile 2	50/44079	0.52 (0.37, 0.74)		0.45 (0.32, 0.64)		0.59 (0.42, 0.84)	
Quintile 3	46/39434	0.54 (0.38, 0.77)	<0.001	0.43 (0.30, 0.61)	<0.001	0.60 (0.42, 0.86)	<0.001
Quintile 4	33/41873	0.36 (0.24, 0.54)		0.28 (0.19, 0.43)		0.44 (0.29, 0.66)	
Quintile 5	37/40594	0.42 (0.29, 0.62)		0.30 (0.20, 0.44)		0.54 (0.36, 0.80)	

Z-score	254/206463	0.67 (0.59, 0.76)	<0.001	0.61 (0.53, 0.69)	<0.001	0.75 (0.66, 0.86)	<0.001
DASH score							
Quintile 1	81/46191	1 (Ref.)		1 (Ref.)		1 (Ref.)	
Quintile 2	43/29724	0.82 (0.57, 1.19)		0.70 (0.48, 1.01)		0.94 (0.65, 1.37)	
Quintile 3	69/52116	0.75 (0.55, 1.04)	<0.001	0.58 (0.42, 0.80)	<0.001	0.91 (0.65, 1.26)	0.001
Quintile 4	32/31590	0.58 (0.38, 0.87)		0.43 (0.28, 0.64)		0.74 (0.48, 1.12)	
Quintile 5	29/46842	0.35 (0.23, 0.54)		0.25 (0.16, 0.38)		0.48 (0.31, 0.74)	
Z-score	254/206463	0.72 (0.64, 0.82)	<0.001	0.62 (0.54, 0.70)	<0.001	0.81 (0.71, 0.92)	0.001
EAT-Lancet score							
Quintile 1	95/44399	1 (Ref.)		1 (Ref.)		1 (Ref.)	
Quintile 2	45/35129	0.60 (0.42, 0.85)		0.54 (0.38, 0.76)		0.65 (0.46, 0.93)	
Quintile 3	44/39723	0.52 (0.36, 0.74)	<0.001	0.44 (0.31, 0.62)	<0.001	0.61 (0.42, 0.87)	<0.001
Quintile 4	37/50390	0.34 (0.23, 0.50)		0.28 (0.19, 0.41)		0.44 (0.30, 0.65)	
Quintile 5	33/36822	0.42 (0.28, 0.62)		0.34 (0.23, 0.50)		0.55 (0.37, 0.83)	
Z-score	254/206463	0.65 (0.58, 0.74)	<0.001	0.63 (0.55, 0.71)	<0.001	0.77 (0.68, 0.88)	<0.001
MIND score							
Quintile 1	78/40525	1 (Ref.)		1 (Ref.)		1 (Ref.)	
Quintile 2	66/40865	0.84 (0.60, 1.16)		0.76 (0.54, 1.05)		0.97 (0.70, 1.36)	
Quintile 3	53/46329	0.59 (0.42, 0.84)	<0.001	0.51 (0.36, 0.72)	<0.001	0.75 (0.53, 1.07)	<0.001
Quintile 4	30/38613	0.40 (0.26, 0.61)		0.34 (0.22, 0.51)		0.57 (0.37, 0.87)	
Quintile 5	27/40131	0.35 (0.22, 0.54)		0.27 (0.18, 0.42)		0.49 (0.31, 0.78)	
Z-score	254/206463	0.62 (0.54, 0.70)	<0.001	0.60 (0.53, 0.68)	<0.001	0.76 (0.67, 0.87)	<0.001
Overall PDI score							
Quintile 1	58/41525	1 (Ref.)	0.050	1 (Ref.)	0.001	1 (Ref.)	0.184
Quintile 2	47/37372	0.90 (0.61, 1.32)		0.84 (0.57, 1.23)		0.96 (0.65, 1.42)	

Quintile 3	63/44877	1.00 (0.70, 1.43)		0.88 (0.62, 1.26)		1.10 (0.76, 1.59)	
Quintile 4	47/40539	0.82 (0.56, 1.21)		0.70 (0.47, 1.02)		0.89 (0.60, 1.32)	
Quintile 5	39/42150	0.66 (0.44, 0.99)		0.53 (0.35, 0.79)		0.74 (0.49, 1.13)	
Z-score	254/206463	0.87 (0.77, 0.99)	0.029	0.81 (0.71, 0.91)	0.001	0.90 (0.80, 1.03)	0.123
Healthful PDI score							
Quintile 1	67/41574	1 (Ref.)		1 (Ref.)		1 (Ref.)	
Quintile 2	67/44376	0.94 (0.67, 1.32)		0.83 (0.59, 1.17)		0.99 (0.70, 1.40)	
Quintile 3	44/37624	0.73 (0.50, 1.06)	0.001	0.61 (0.42, 0.90)	<0.001	0.80 (0.54, 1.18)	0.050
Quintile 4	33/42166	0.49 (0.32, 0.74)		0.39 (0.26, 0.60)		0.56 (0.36, 0.86)	
Quintile 5	43/40723	0.66 (0.45, 0.96)		0.53 (0.36, 0.77)		0.83 (0.55, 1.26)	
Z-score	254/206463	0.80 (0.71, 0.91)	<0.001	0.73 (0.65, 0.83)	<0.001	0.86 (0.75, 0.99)	0.033
Unhealthful PDI score							
Quintile 1	38/40958	1 (Ref.)		1 (Ref.)		1 (Ref.)	
Quintile 2	52/46503	1.20 (0.79, 1.83)		1.36 (0.90, 2.07)		1.41 (0.92, 2.16)	
Quintile 3	46/34414	1.44 (0.93, 2.00)	0.036	1.62 (1.06, 2.50)	<0.001	1.62 (1.04, 2.52)	0.004
Quintile 4	64/43973	1.56 (1.05, 2.33)		1.97 (1.32, 2.94)		1.85 (1.21, 2.82)	
Quintile 5	54/40615	1.43 (0.94, 2.16)		1.89 (1.25, 2.87)		1.85 (1.17, 2.93)	
Z-score	254/206463	1.16 (1.03, 1.31)	0.015	1.30 (1.15, 1.48)	<0.001	1.29 (1.12, 1.49)	<0.001

Each dietary pattern score was presented as the merging-sex specific quintiles. Model 1: unadjusted model. Model 2 was adjusted for age, sex, and ethnicity. Model 3 included further adjustments for Townsend deprivation scores, educational attainment, physical activity levels, smoking status, alcohol consumption, total energy intake, BMI, CVD, hypertension, T2DM, respiratory diseases, occupation-related breathing problems, and air pollution factors: including, NO, NO_2, $PM_{2.5}$, $PM_{2.5\text{-}10}$, PM_{10}. Abbreviations: BMI, body mass index; CVD, cardiovascular disease; PM, Particulate matter; T2DM, type 2 diabetes mellitus; NO, Nitrogen oxide; NO_2, Nitrogen dioxide.

Table S10 Net reclassification improvement (NRI), integrated discrimination improvement (IDI), and area under the ROC curve (AUC) for the risk of Chronic obstructive pulmonary disease (COPD) associated with multiple dietary patterns.

	Net reclassification improvement (NRI)		Integrated discrimination improvement (IDI)		Area under the ROC curve (AUC)		
	NRI (95% CI), %	*P* value	IDI (95% CI), %	*P* value	AUC (×100)	ΔAUC (95% CI), %	*P* value
Reference model	Ref		Ref		86.29	Ref	
Reference model +							
AHA diet	11.89 (7.910, 15.88)	<0.001	0.086 (0.050, 0.130)	<0.001	86.36	0.068 (0.002, 0.134)	0.043
AMED	10.37 (6.390, 14.35)	<0.001	0.073 (0.030, 0.110)	<0.001	86.38	0.084 (0.015, 0.152)	0.017
AHEI-2010	7.703 (3.710, 11.69)	<0.001	0.056 (0.020, 0.090)	0.002	86.36	0.065 (0.009, 0.122)	0.024
DASH	9.196 (5.210, 13.19)	<0.001	0.066 (0.020, 0.110)	0.002	86.38	0.088 (0.022, 0.154)	0.009
EAT-Lancet	10.24 (6.250, 14.22)	<0.001	0.062 (0.020, 0.100)	0.002	86.37	0.074 (0.013, 0.136)	0.018
MIND	10.89 (6.910, 18.87)	<0.001	0.074 (0.030, 0.110)	<0.001	86.37	0.076 (0.008, 0.144)	0.029
Overall PDI	2.144 (-1.850, 6.140)	0.293	0.009 (-0.010, 0.020)	0.276	86.31	0.020 (0.006, 0.047)	0.131
Healthful PDI	7.610 (3.630, 11.59)	<0.001	0.025 (0.000, 0.050)	0.050	86.35	0.061 (0.014, 0.109)	0.012
Unhealthful PDI	10.04 (6.060, 14.03)	<0.001	0.052 (0.018, 0.090)	0.005	86.37	0.076 (0.018, 0.135)	0.011

The reference model included the following factors: age, sex, ethnicity, Townsend index, education, BMI, physical activity, smoking, alcohol, total energy intake, CVD, T2DM, hypertension, other respiratory diseases, occupation-related respiratory problems and all air pollution factors. Abbreviations: AHA diet, American Heart Association diet; AHEI, Alternate Healthy Eating Index; AMED: alternate Mediterranean diet; DASH, Dietary Approaches to Stop Hypertension; MIND, Mediterranean Dietary Approaches to Stop Hypertension Intervention for Neurodegenerative Delay diet; PDI, plant-based diet index; BMI, body mass index; CVD, cardiovascular disease; T2DM, type 2 diabetes mellitus.

Table S11 Hazard ratios (HR) and 95% confidence intervals (CI) for the risk of Chronic obstructive pulmonary disease (COPD) with multiple dietary patterns (Z-scores) from sensitivity analyses.

Case/N	Sensitivity Analyze 1		Sensitivity Analyze 2		Sensitivity Analyze 3		Sensitivity Analyze 4	
	2133/206146		1283/125338		1136/127174		668/132750	
	HR (95%CI)	*P* value	HR (95%CI)	*P* value	HR (95%CI)	*P* value	HR (95%CI)	*P* value
AHA diet score	0.87 (0.83, 0.92)	<0.001	0.87 (0.82, 0.92)	<0.001	0.85 (0.80, 0.91)	<0.001	0.87 (0.80, 0.94)	0.001
AMED score	0.86 (0.82, 0.90)	<0.001	0.90 (0.85, 0.95)	0.001	0.84 (0.79, 0.90)	<0.001	0.86 (0.79, 0.94)	0.001
AHEI score	0.89 (0.85, 0.93)	<0.001	0.90 (0.84, 0.95)	<0.001	0.87 (0.82, 0.93)	<0.001	0.84 (0.78, 0.94)	<0.001
DASH score	0.88 (0.84, 0.92)	<0.001	0.88 (0.83, 0.94)	<0.001	0.84 (0.79, 0.89)	<0.001	0.84 (0.78, 0.91)	<0.001
EAT-Lancet score	0.89 (0.85, 0.93)	<0.001	0.90 (0.85, 0.96)	<0.001	0.86 (0.80, 0.91)	<0.001	0.92 (0.85, 0.99)	0.024
MIND score	0.87 (0.84, 0.91)	<0.001	0.89 (0.84, 0.94)	<0.001	0.85 (0.80, 0.91)	<0.001	0.87 (0.80, 0.94)	0.001
Overall PDI score	0.95 (0.91, 0.99)	0.013	0.96 (0.90, 1.02)	0.171	0.93 (0.88, 0.99)	0.017	0.93 (0.86, 1.01)	0.080
Healthful PDI score	0.90 (0.86, 0.95)	<0.001	0.86 (0.81, 0.92)	<0.001	0.88 (0.83, 0.94)	<0.001	0.91 (0.84, 0.99)	0.031
Unhealthful PDI score	1.13 (1.08, 1.19)	<0.001	1.14 (1.07, 1.22)	<0.001	1.18 (1.11, 1.26)	<0.001	1.16 (1.06, 1.26)	0.001

Sensitivity Analysis 1: excluding those participants occurred incident COPD within the first two years of follow-up. Sensitivity Analysis 2: including those participants, they had at least twice 24h dietary records. Sensitivity Analysis 3: excluding those participants who had respiratory diseases or records of occupation-related breathing problems at baseline. Sensitivity Analyze 4: including those participants had the records of FEV1/FVC, and their ratio more than 0.70. Models were adjusted for age, sex, and ethnicity, Townsend deprivation scores, educational attainment, physical activity levels, smoking status, alcohol consumption, total energy intake, BMI, CVD, hypertension, T2DM, respiratory diseases, occupation-related breathing problems, and air pollution factors: including, NO, NO_2, $PM_{2.5}$, $PM_{2.5-10}$, PM_{10}. Abbreviations: BMI, body mass index; CVD, cardiovascular disease; PM, Particulate matter; T2DM, type 2 diabetes mellitus; NO, Nitrogen oxide; NO_2, Nitrogen dioxide.

Table S12 Hazard ratios (HR) and 95% confidence intervals (CI) for stratified analyses and modified effects, with interaction assessed on a multiplicative scale, examining the association for the risk of COPD with Z-scores of AHA diet, AMED, AHEI-2010, DASH, or EAT-Lancet score.

	Case/Total	AHA diet score	AMED score	AHEI-2010 score	DASH score	EAT-Lancet score
Age						
< 57 years	481/98085	0.82 (0.74, 0.90)	0.80 (0.72, 0.89)	0.84 (0.76, 0.92)	0.86 (0.78, 0.94)	0.81 (0.74, 0.89)
≥ 57 years	1969/108378	0.89 (0.85, 0.93)	0.88 (0.84, 0.93)	0.91 (0.87, 0.96)	0.89 (0.85, 0.93)	0.91 (0.87, 0.95)
P for interaction		**0.006****	0.191	0.124	0.140	**0.028***
White race						
No	63/9217	1.09 (0.82, 1.45)	1.01 (0.76, 1.33)	1.12 (0.87, 1.43)	1.07 (0.84, 1.37)	1.06 (0.83, 1.36)
Yes	2387/197246	0.87 (0.83, 0.91)	0.87 (0.83, 0.91)	0.89 (0.86, 0.93)	0.88 (0.84, 0.92)	0.89 (0.85, 0.92)
P for interaction		0.237	0.101	0.046	0.064	0.096
Sex						
Female	1071/113872	0.92 (0.86, 0.98)	0.90 (0.84, 0.96)	0.94 (0.88, 1.00)	0.89 (0.83, 0.94)	0.91 (0.85, 0.96)
Male	1379/92591	0.84 (0.79, 0.89)	0.84 (0.79, 0.89)	0.86 (0.81, 0.91)	0.88 (0.83, 0.93)	0.88 (0.83, 0.93)
P for interaction		0.079	0.847	0.216	0.433	0.821
BMI						
< 26.2 kg/m^2	980/103511	0.86 (0.80, 0.92)	0.87 (0.81, 0.93)	0.89 (0.83, 0.95)	0.86 (0.80, 0.91)	0.86 (0.80, 0.92)
≥ 26.2 kg/m^2	1470/102952	0.89 (0.84, 0.94)	0.87 (0.82, 0.92)	0.90 (0.85, 0.95)	0.91 (0.86, 0.95)	0.91 (0.86, 0.96)
P for interaction		0.425	0.140	0.293	**0.001****	**0.013***
Smoking status						
Never	475/116866	1.01 (0.91, 1.12)	0.98 (0.89, 1.08)	0.99 (0.90, 1.09)	0.98 (0.89, 1.08)	1.01 (0.92, 1.11)
Previous	1257/73100	0.84 (0.79, 0.89)	0.84 (0.79, 0.89)	0.87 (0.83, 0.93)	0.87 (0.82, 0.92)	0.86 (0.81, 0.91)
Current	707/15956	0.86 (0.79, 0.93)	0.86 (0.79, 0.94)	0.88 (0.82, 0.95)	0.86 (0.8, 0.93)	0.88 (0.81, 0.95)
P for interaction		0.793	0.212	0.546	0.208	0.114

Alcohol consumption						
Never	84/6609	1.08 (0.86, 1.37)	1.28 (1.01, 1.63)	1.15 (0.92, 1.43)	1.05 (0.84, 1.30)	1.09 (0.87, 1.35)
Previous	175/6176	0.97 (0.82, 1.14)	0.97 (0.81, 1.15)	0.99 (0.85, 1.16)	0.97 (0.84, 1.12)	0.97 (0.84, 1.14)
Current	2189/193485	0.86 (0.82, 0.90)	0.85 (0.81, 0.89)	0.88 (0.84, 0.92)	0.87 (0.83, 0.91)	0.87 (0.84, 0.91)
P for interaction		0.141	**0.017***	0.073	0.150	0.068
Physical activity						
Low	506/32194	0.88 (0.80, 0.97)	0.89 (0.80, 0.98)	0.88 (0.81, 0.97)	0.86 (0.79, 0.94)	0.88 (0.80, 0.96)
Moderate	761/74206	0.92 (0.85, 0.99)	0.91 (0.84, 0.98)	0.91 (0.85, 0.98)	0.93 (0.87, 1.01)	0.91 (0.85, 0.99)
High	680/68641	0.82 (0.76, 0.89)	0.87 (0.80, 0.95)	0.88 (0.81, 0.95)	0.84 (0.78, 0.91)	0.88 (0.80, 0.96)
P for interaction		0.410	**0.045***	0.394	0.340	0.493
CVD						
No	1977/195263	0.87 (0.83, 0.91)	0.86 (0.82, 0.91)	0.89 (0.85, 0.93)	0.87 (0.83, 0.91)	0.88 (0.84, 0.92)
Yes	473/11200	0.91 (0.82, 1.00)	0.88 (0.79, 0.98)	0.92 (0.83, 1.01)	0.95 (0.87, 1.05)	0.92 (0.84, 1.01)
P for interaction		0.613	0.731	0.698	0.132	0.342
T2DM						
No	2216/198797	0.88 (0.84, 0.92)	0.87 (0.83, 0.91)	0.90 (0.86, 0.94)	0.88 (0.84, 0.92)	0.89 (0.85, 0.93)
Yes	234/7666	0.87 (0.76, 0.99)	0.84 (0.72, 0.98)	0.88 (0.77, 1.01)	0.92 (0.80, 1.05)	0.90 (0.78, 1.03)
P for interaction		0.418	0.900	0.426	0.845	0.745
Hypertension						
No	706/100038	0.87 (0.81, 0.95)	0.87 (0.80, 0.95)	0.90 (0.83, 0.97)	0.83 (0.77, 0.90)	0.86 (0.80, 0.93)
Yes	1744/106425	0.88 (0.83, 0.92)	0.87 (0.82, 0.92)	0.90 (0.85, 0.94)	0.91 (0.86, 0.95)	0.90 (0.86, 0.95)
P for interaction		0.675	0.834	0.883	0.068	0.254

All Models were adjusted for age, sex, ethnicity, Townsend index, education, BMI, physical activity, smoking, alcohol, total energy intake, CVD, T2DM, hypertension, other respiratory diseases, occupation-related respiratory problems and all air pollution factors. Abbreviations: CVD, cardiovascular disease; T2DM, type 2 diabetes mellitus.

Table S13 Hazard ratios (HR) and 95% confidence intervals (CI) for stratified analyses and modified effects, with interaction assessed on a multiplicative scale, examining the association for the risk of COPD with Z-scores of MIND, Overall PDI, Healthful PDI, or Unhealthful PDI score.

	Case/Total	MIND score	Overall PDI score	Healthful PDI score	Unhealthful PDI score
Age (years)					
< 57	481/98085	0.86 (0.78, 0.95)	0.94 (0.86, 1.03)	0.88 (0.80, 0.97)	1.19 (1.08, 1.32)
≥ 57	1969/108378	0.88 (0.84, 0.92)	0.96 (0.91, 1.00)	0.92 (0.87, 0.96)	1.12 (1.06, 1.18)
P for interaction		0.264	0.999	0.052	0.324
White race					
No	63/9217	1.01 (0.77, 1.31)	1.12 (0.87, 1.44)	0.94 (0.73, 1.22)	1.06 (0.80, 1.4)
Yes	2387/197246	0.87 (0.84, 0.91)	0.95 (0.91, 0.99)	0.91 (0.87, 0.95)	1.13 (1.08, 1.19)
P for interaction		0.242	0.037	0.928	0.353
Sex					
Female	1071/113872	0.88 (0.83, 0.94)	0.99 (0.93, 1.05)	0.93 (0.88, 1.00)	1.15 (1.07, 1.23)
Male	1379/92591	0.87 (0.82, 0.92)	0.92 (0.87, 0.97)	0.89 (0.84, 0.94)	1.12 (1.05, 1.19)
P for interaction		0.512	0.547	0.253	0.095
BMI (kg/m^2)					
< 26.2	980/103511	0.89 (0.83, 0.95)	0.97 (0.91, 1.04)	0.87 (0.81, 0.94)	1.17 (1.09, 1.26)
≥ 26.2	1470/102952	0.87 (0.83, 0.92)	0.95 (0.90, 1.00)	0.93 (0.88, 0.99)	1.11 (1.05, 1.18)
P for interaction		0.257	0.510	0.304	0.039
Smoking status					
Never	475/116866	1.01 (0.92, 1.11)	1.00 (0.91, 1.10)	0.93 (0.84, 1.03)	1.07 (0.97, 1.19)
Previous	1257/73100	0.84 (0.8, 0.89)	0.91 (0.86, 0.97)	0.91 (0.86, 0.97)	1.15 (1.08, 1.23)
Current	707/15956	0.86 (0.79, 0.93)	0.94 (0.87, 1.01)	0.91 (0.84, 0.99)	1.13 (1.04, 1.23)

P for interaction		0.854	0.410	0.920	0.584
Alcohol consumption					
Never	84/6609	1.15 (0.92, 1.42)	0.94 (0.75, 1.19)	1.01 (0.81, 1.27)	0.96 (0.76, 1.22)
Previous	175/6176	0.98 (0.84, 1.14)	1.11 (0.94, 1.30)	1.00 (0.85, 1.17)	1.07 (0.90, 1.26)
Current	2189/193485	0.86 (0.82, 0.90)	0.94 (0.90, 0.98)	0.90 (0.86, 0.94)	1.15 (1.09, 1.20)
P for interaction		0.050	0.275	0.215	0.563
Physical activity					
Low	506/32194	0.91 (0.82, 1.00)	0.91 (0.83, 1.00)	0.92 (0.83, 1.01)	1.13 (1.02, 1.25)
Moderate	761/74206	0.88 (0.81, 0.94)	0.97 (0.90, 1.04)	0.92 (0.85, 1.00)	1.06 (0.97, 1.15)
High	680/68641	0.85 (0.79, 0.92)	0.96 (0.89, 1.04)	0.89 (0.82, 0.97)	1.17 (1.08, 1.28)
P for interaction		0.606	0.910	0.345	0.203
CVD					
No	1977/195263	0.87 (0.83, 0.91)	0.95 (0.91, 0.99)	0.90 (0.85, 0.94)	1.14 (1.09, 1.20)
Yes	473/11200	0.90 (0.81, 0.99)	0.96 (0.88, 1.06)	0.96 (0.87, 1.06)	1.09 (0.98, 1.21)
P for interaction		0.541	0.781	0.355	0.640
T2DM					
No	2216/198797	0.88 (0.84, 0.92)	0.95 (0.91, 1.00)	0.91 (0.87, 0.95)	1.12 (1.07, 1.18)
Yes	234/7666	0.88 (0.77, 1.01)	0.94 (0.82, 1.07)	0.90 (0.79, 1.03)	1.19 (1.03, 1.38)
P for interaction		0.975	0.898	0.606	0.605
Hypertension					
No	706/100038	0.90 (0.83, 0.97)	0.97 (0.90, 1.04)	0.88 (0.82, 0.96)	1.17 (1.08, 1.28)
Yes	1744/106425	0.87 (0.83, 0.91)	0.95 (0.90, 1.00)	0.92 (0.88, 0.97)	1.12 (1.06, 1.18)
P for interaction		0.594	0.609	0.390	0.398

All Models were adjusted for age, sex, ethnicity, Townsend index, education, BMI, physical activity, smoking, alcohol, total energy intake, CVD, T2DM, hypertension, other respiratory diseases, occupation-related respiratory problems and all air pollution factors. Abbreviations: CVD, cardiovascular disease; T2DM, type 2 diabetes mellitus.

e-FIGURES

Figure S1 Dietary factors of dietary patterns.

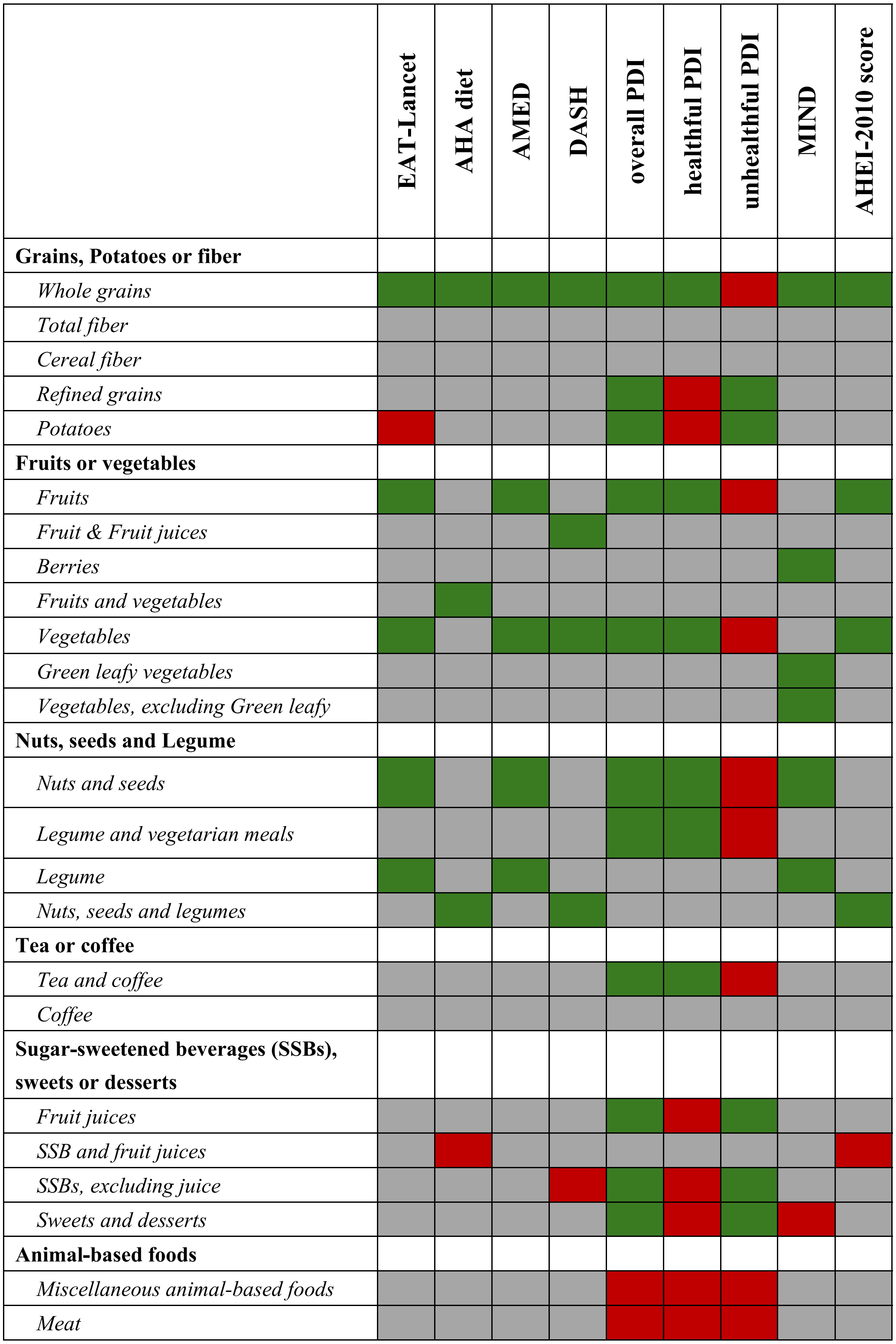

Red & processed meat			Red	Red				Red	Red
Processed meat		Red							
Beef and lamb	Red								
Pork	Red								
Poultry	Red							Green	
**Fish or seafood**									
Fish	Green	Green	Green					Green	
Fish and other seafood					Red	Red	Red		
Oily fish					Red	Red	Red		
**Eggs or Dairy**									
Eggs	Red				Red	Red	Red		
Dairy	Red				Red	Red	Red		
low fatty dairy				Green					
Cheese								Red	
**Fast foods or Added sugar**									
Added sugar	Red								
Ultra-processed foods									
Fried/fast food								Red	
Glycemic index, GI									
**Fats or fat acids**									
Trans-fat acids									Red
MUFA: SFA ratio			Green					Green	
PUFA			Green					Green	
PUFA:SFA ratio									
SFA		Red							
(MUFA+PUFA)/SFA ratio	Green								
EPA & DHA									Green
PUFA excluding EPA & DHA									Green
Butter and stick margarine								Red	
Animal fat					Red	Red	Red		
**Alcohol**									
Alcohol			Yellow						Yellow
Wine								Yellow	
**Breastfed as a baby**									
**Sodium (mg/d)**		Red		Red					Red

Red: lower points were assigned to higher intakes; green: higher points were assigned to higher intakes; yellow: higher points were assigned to moderate intake.

Figure S2 Potential non-linear relationship between multiple dietary patterns scores and the risk of COPD-caused mortality using restricted cubic spline regression.

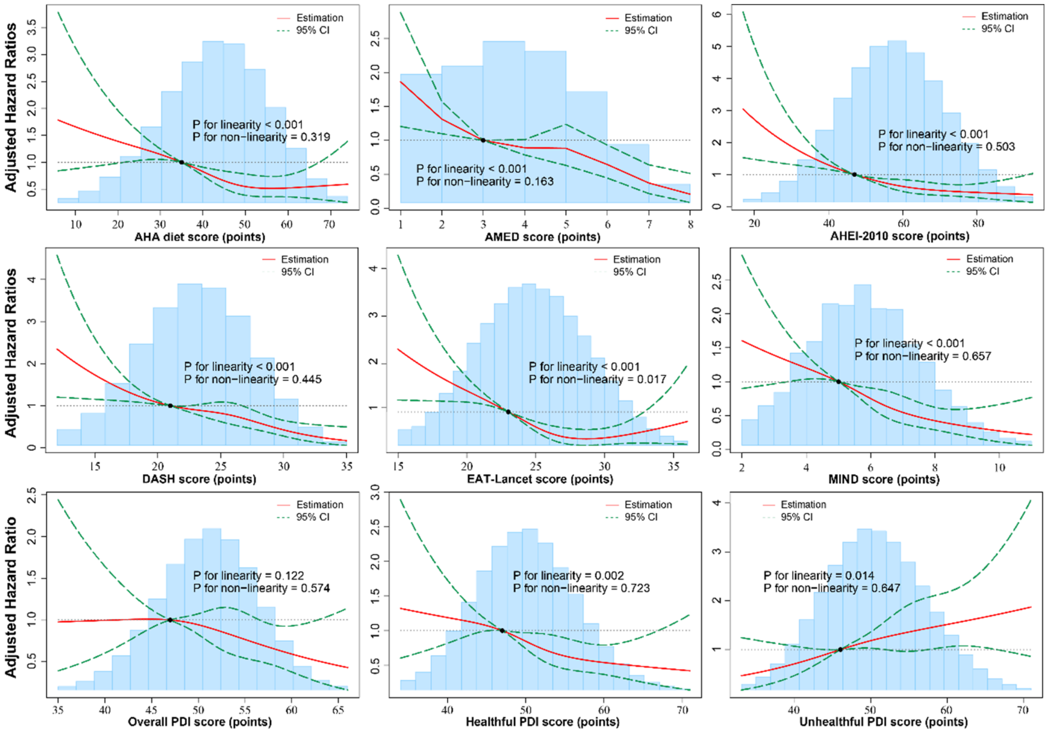

The reference point is the 25th percentile of the reference group from categorical analysis with 4 knots. All Models were adjusted for age, sex, ethnicity, Townsend index, education, BMI, physical activity, smoking, alcohol, total energy intake, CVD, T2DM, hypertension, other respiratory diseases, occupation-related respiratory problems and all air pollution factors. Abbreviations: BMI, body mass index; CVD, cardiovascular disease; T2DM, type 2 diabetes mellitus.

e-REFERENCES

1. Rehm CD, Penalvo JL, Afshin A, Mozaffarian D. Dietary Intake Among US Adults, 1999-2012. JAMA 2016;315(23):2542-53. doi: 10.1001/jama.2016.7491.
2. Lloyd-Jones DM, Hong Y, Labarthe D, Mozaffarian D, Appel LJ, Van Horn L, Greenlund K, Daniels S, Nichol G, Tomaselli GF, et al. Defining and setting national goals for cardiovascular health promotion and disease reduction: the American Heart Association's strategic Impact Goal through 2020 and beyond. Circulation 2010;121(4):586-613. doi: 10.1161/CIRCULATIONAHA.109.192703.
3. Fung TT, Rexrode KM, Mantzoros CS, Manson JE, Willett WC, Hu FB. Mediterranean diet and incidence of and mortality from coronary heart disease and stroke in women. Circulation 2009;119(8):1093-100. doi: 10.1161/CIRCULATIONAHA.108.816736.
4. Chiuve SE, Fung TT, Rimm EB, Hu FB, McCullough ML, Wang M, Stampfer MJ, Willett WC. Alternative dietary indices both strongly predict risk of chronic disease. J Nutr 2012;142(6):1009-18. doi: 10.3945/jn.111.157222.
5. Fung TT, Chiuve SE, McCullough ML, Rexrode KM, Logroscino G, Hu FB. Adherence to a DASH-style diet and risk of coronary heart disease and stroke in women. Arch Intern Med 2008;168(7):713-20. doi: 10.1001/archinte.168.7.713.
6. Willett W, Rockstrom J, Loken B, Springmann M, Lang T, Vermeulen S, Garnett T, Tilman D, DeClerck F, Wood A, et al. Food in the anthropocene: the EAT-lancet commission on healthy diets from sustainable food systems. Lancet 2019;393(10170):447-92. doi: 10.1016/S0140-6736(18)31788-4.
7. Stubbendorff A, Sonestedt E, Ramne S, Drake I, Hallström E, Ericson U. Development of an EAT-Lancet index and its relation to mortality in a Swedish population. The American Journal of Clinical Nutrition 2022;115(3):705-16. doi: 10.1093/ajcn/nqab369.
8. Lu X, Wu L, Shao L, Fan Y, Pei Y, Lu X, Borné Y, Ke C. Adherence to the EAT-Lancet diet and incident depression and anxiety. Nature Communications 2024;15(1). doi: 10.1038/s41467-024-49653-8.
9. Morris MC, Tangney CC, Wang Y, Sacks FM, Bennett DA, Aggarwal NT. MIND diet associated with reduced incidence of Alzheimer's disease. Alzheimer's & Dementia 2015;11(9):1007-14. doi: 10.1016/j.jalz.2014.11.009.
10. Satija A, Bhupathiraju SN, Rimm EB, Spiegelman D, Chiuve SE, Borgi L, Willett WC, Manson JE, Sun Q, Hu FB. Plant-Based Dietary Patterns and Incidence of Type 2 Diabetes in US Men and Women: Results from Three Prospective Cohort Studies. PLoS Med 2016;13(6):e1002039. doi: 10.1371/journal.pmed.1002039.
11. Sullivan VK, Kim H, Caulfield LE, Steffen LM, Selvin E, Rebholz CM. Plant-Based Dietary Patterns and Incident Diabetes in the Atherosclerosis Risk in Communities (ARIC) Study. Diabetes Care 2024;47(5):803-9. doi: 10.2337/dc23-2013.
12. Stockholm Resilience Centre. Nordic food systems for improved health and sustainability. Baseline assessment to inform transformation. 2019.
